# Melatonin as a protector against endocrine disruption across tissues: A systematic review

**DOI:** 10.14814/phy2.71049

**Published:** 2026-08-10

**Authors:** Clenie Isingizwe, Gaudence Ndinganire, Abdullateef Isiaka Alagbonsi

**Affiliations:** ^1^ Department of Physiology, School of Medicine and Pharmacy, College of Medicine and Health Sciences University of Rwanda Huye Rwanda

**Keywords:** endocrine‐disrupting chemicals, hormonal regulation, melatonin, mitochondrial protection, oxidative stress

## Abstract

Endocrine‐disrupting chemicals (EDCs) are widespread environmental pollutants that interfere with hormonal homeostasis and contribute to multisystem toxicity. This systematic review aimed to synthesize available evidence on the protective effect of melatonin against EDC‐induced toxicity across tissues. English language articles published between January 2000 and December 2025 were searched on PubMed, Web of Science, and Wiley Online. Eligible studies evaluated the protective or antagonistic effects of melatonin against EDC‐induced toxicity in humans, mammals, or in vitro models. Data on study characteristics, EDC exposure, melatonin intervention, affected tissues, outcomes, and mechanisms were extracted, synthesized, and reported in accordance with the PRISMA 2020 guidelines. Sixty‐one studies met the inclusion criteria. Melatonin exhibited protective effects against diverse EDCs, including bisphenols, phthalates, heavy metals, pesticides, and microplastics. The principal mechanisms identified were antioxidant and free‐radical scavenging activity, modulation of hormone receptors, regulation of gene and protein expression, stabilization of endocrine axes, mitochondrial protection, and epigenetic modulation. These effects were observed across multiple organ systems, particularly the reproductive, neuroendocrine, cardiovascular, adrenal, and immune systems. Melatonin protects against EDC‐induced multisystem toxicities by preserving endocrine signaling and coordinating cellular defense mechanisms across tissues, thereby holding therapeutic potential.

## Graphical abstract

Graphical Abstract showing the biological mechanisms by which melatonin protects tissues against endocrine disruption.

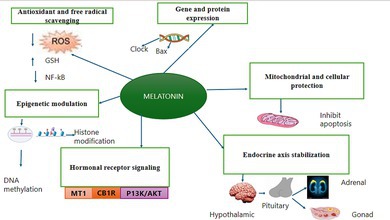

## 1 INTRODUCTION

Endocrine disrupting chemicals (EDCs), such as plasticizers like bisphenol A (BPA) and phthalates (di‐(2‐ethylhexyl) phthalate, DEHP), heavy metals like cadmium (Cd) and lead (Pb), and persistent organic pollutants, are widely present in the environment as a result of the world's increasing industrialization. These chemicals are infamous for their capacity to cause multisystemic toxicity by interfering with hormonal signaling. The development of oxidative stress, which is marked by an increase in reactive oxygen species (ROS) and lipid peroxidation, is one of the main mechanisms of damage caused by EDCs. For example, exposure to BPA has been demonstrated to considerably lower antioxidant defenses and raise oxidative markers in a variety of tissues (Akçay et al., 2020).

Melatonin, a highly conserved indoleamine that is mostly synthesized and secreted by the pineal gland (Reiter, 2010), is widely produced across species, ranging from prokaryotes (archaea, cyanobacteria, and bacteria), unicellular eukaryotes, multicellular organisms like plants, fungi, and all animals (Back, 2021; Kim et al., 2024) and thus, estimated to be 2.5 billion years old (Reiter et al., 2017). Beyond the pineal gland, melatonin is now known to be synthesized in many extra‐pineal tissues, like the skin, ovaries, retina, bone marrow, immune cells, gastrointestinal tract (GIT), and placenta, where it participates in the local regulation of cell function, oxidative defense, metabolism, and immunological responses, among others, via autocrine and paracrine signaling (Ndinganire et al., 2026). Melatonin also modulates the physiological and biochemical processes in plants, though phytomelatonin‐deficient *Coffee arabica* has been documented in Rwanda (Rurangwa et al., 2025).

The protective effect of melatonin against the toxic effects of EDCs has been reported in various systems of the body. In the endocrine system, EDCs like Cd, BPA, nonylphenol (NP), and polycyclic aromatic hydrocarbons (PAHs) affect thyroid and adrenal functions (Jiménez‐Ortega et al., 2012). By restoring redox balance, resetting circadian clock genes (Clock, Bmal1), and safeguarding endocrine organs, melatonin lessens these effects (Farag et al., 2024; Olukole et al., 2018). In the reproductive system, EDCs such as Cd, BPA, decabromodiphenyl ether (BDE‐209), DEHP, bisphenol S (BPS), tetrabromobisphenol A (TBBPA), and oxybenzone are extremely dangerous to both male and female reproductive processes, as they cause reduced sperm quality, abnormal oocyte maturation, compromised ovarian and testicular structure, and dysregulated reproductive hormones (Chouchene et al., 2025; Shi et al., 2025; Venditti et al., 2021a). Melatonin antagonizes these toxicities by enhancing antioxidant defenses, restoring mitochondrial and spindle function, modulating autophagy/mitophagy pathways, and normalizing key reproductive proteins such as disheveled‐associated activator of morphogenesis 1 (DAAM1), prolyl endopeptidase (PREP), follicle‐stimulating hormone receptor (FSHR), and connexin 43 (Akarca‐Dizakar et al., 2020; Lin et al., 2025). In the hippocampus, frontal cortex, and cerebrum, EDCs like BPA, Cd, Pb, and NP cause oxidative stress, neuroinflammation, cognitive impairments, apoptosis, and neuronal damage (Ishtiaq et al., 2021; Omeiza et al., 2021), and melatonin provides neuroprotection by scavenging ROS, replenishing antioxidant enzymes, modifying apoptotic proteins p53, p53 upregulated modulator of apoptosis (PUMA), and reducing neuroinflammatory reactions (Ishtiaq et al., 2021). Similarly, the toxic effects of EDCs (Chukwu et al., 2025), especially on the cardiovascular (Sarihan et al., 2015), respiratory, renal (Karaoz et al., 2002), gastrointestinal (Ebrahimi et al., 2021), and musculoskeletal (Turker Yavas et al., 2024) systems, have been reported.

As induction of oxidative stress is a central mechanism for toxic effects induced by EDCs across tissues, agents with antioxidative actions hold a very promising therapeutic potential in combating EDC‐induced toxic effects widely reported in the literature. Interestingly, melatonin is well‐known to scavenge free radicals and suppress oxidative stress in many disease conditions (Abdulrahim et al., 2021; Alagbonsi et al., 2016, 2020; Alagbonsi & Olayaki, 2017, 2018; Maliki et al., 2021; Olayaki et al., 2016, 2017, 2018; Oluwasola, 2026; Omeiza et al., 2021). However, melatonin is not currently recognized as a drug or supplement that can manage disorders arising from EDC exposures, despite convincing evidence from several studies across body organ systems. To draw the attention of scientists and healthcare providers to this important therapeutic potential of melatonin, a well‐designed systematic review is needed. This study aimed to synthesize available data on how melatonin counteracts endocrine disruption across body tissues.

## 2 METHODOLOGY

### 2.1 Study design

This study was designed as a systematic review reported in accordance with the Preferred Reporting Items for Systematic Reviews and Meta‐Analyses (PRISMA) 2020 guidelines (Page et al., 2021), as adopted in some previous systematic reviews (Hakizimana et al., 2025; Hakizimana & Alagbonsi, 2025, 2026; Hakizimana, Ntakirutimana, et al., 2026; Hakizimana, Olubiyi, & Alagbonsi, 2026; Izabayo et al., 2026; Mucumbitsi et al., 2026). The PRISMA checklist can be found in the File S1.

### 2.2 Search strategy

A comprehensive literature search was conducted across various electronic databases, including PubMed, Web of Science, and Wiley Online, to identify relevant studies. The search was restricted to studies that evaluated the role of melatonin as an antagonist or a protective agent against EDCs across tissues in the biological systems. The search strategy combined Medical Subject Headings (MeSH) and free‐text keywords related to melatonin, endocrine disruptors, and multisystem outcomes. Key search terms included “melatonin,” *“*endocrine disruptors,” “endocrine‐disrupting chemicals,” “bisphenol A,” “phthalates,” “polybrominated diphenyl ethers,” “pesticides,” “perfluoroalkyl substances,” “hormonal disruption,” “oxidative stress,” “reproductive system,” “nervous system,” “metabolic system,” “immune system,” “cardiovascular system,” “respiratory system,” “renal system,” “gastrointestinal system,” and “multisystem toxicity.*”* These terms were combined using Boolean operators (“AND,” “OR”) to capture studies reporting the protective effects of melatonin across multiple tissues and organ systems. The search strings used to retrieve articles from each database are presented in File S2. The initial search strings in File S2 were run through October 2025; a supplementary search using the same terms and databases was subsequently conducted on 2nd January 2026 to capture studies published through December 2025. This supplementary search identified three additional eligible studies published in this window (Chouchene et al., 2025; Oluwasola, 2026; Shi et al., 2025), which were incorporated into the final synthesis, and the final publication date range reported throughout this manuscript reflects this updated search.

### 2.3 Inclusion and exclusion criteria

To ensure the relevance and quality of the selected literature, studies published in English between January 2000 and December 2025 that investigated the protective or modulatory effects of melatonin against EDCs were included, following the Population, Intervention, Comparison, and Outcome (PICO) framework. Eligible studies involved humans, mammals, or in vitro models exposed to recognized EDCs (El Gemayel, 2025) and were required to report outcomes in the tissue of at least one body system, with clearly defined melatonin interventions and measurable biological, molecular, or functional endpoints. Only original research studies were included. Studies were excluded if they were reviews, editorials, meta‐analyses, conference abstracts without full data, or case reports with very small sample sizes. Articles were also excluded if they investigated melatonin without EDC exposure, EDCs without melatonin involvement, or if the effects of melatonin could not be isolated due to combined interventions. Studies published outside the specified time period, those not available in English, and those with insufficient methodological detail, unclear exposure or intervention protocols, or incomplete outcome data were also excluded (Table 1).

**TABLE 1:** Inclusion and exclusion criteria for study selection using the PICO framework.

Criteria type	Inclusion criteria	Exclusion criteria
Language	Articles published in English	Articles published in languages other than English
Publication Date	Studies published between January 2000 and December 2025 (First search ran through October 2025; Second search was conducted on 2nd January 2026 to run through December 2025)	Studies published outside the defined date range
Study Type	Original experimental and observational studies, including randomized controlled clinical trials involving human participants, observational human studies, experimental intervention studies in mammalian animal models, and in vitro experimental studies	Reviews, systematic reviews, meta‐analyses, editorials, commentaries, and conference abstracts without original data
Models Used	Studies involving humans, mammalian animal models, or in vitro cell culture systems	Studies involving plants, microorganisms, or non‐mammalian invertebrate species
Exposure (EDCs)	Exposure to recognized EDCs	Studies without clear exposure to EDCs or involving chemicals not classified as endocrine disruptors
Intervention	Studies that administered or evaluated melatonin as a protective, antagonistic, or modulatory intervention before, during, or after EDC exposure	Studies that involved only EDC exposure without melatonin, or melatonin exposure without EDCs
Focus of Study	Investigated the protective or antagonistic role of melatonin against EDC‐induced toxicity across tissues	Focused on melatonin or EDCs independently without assessing their interaction
Outcomes	Reported system outcomes, including at least one of the following: reproductive, neuroendocrine, metabolic, immune, cardiovascular, or sensory system effects, with measurable biochemical, molecular, functional, or histological endpoints	Studies reporting unrelated outcomes with no endocrine, physiological, or tissue‐specific relevance
Data Quality	Provided clear, extractable, and analyzable quantitative or qualitative data on the effects of melatonin against EDC toxicity	Lacked sufficient outcome data, unclear dosing/exposure protocols, or mixed interventions where melatonin's independent effect could not be isolated
Mechanistic Link	Established a biological, molecular, or physiological mechanism linking melatonin to the mitigation of EDC‐induced effects	General endocrine, antioxidant, or toxicological studies without investigation of melatonin‐specific protective mechanisms against EDCs

### 2.4 Data extraction and analysis

For each eligible study included in this systematic review, the following data were manually extracted from the full text: (i) first author's name and year of publication; (ii) study type and experimental model details (animal model, in vitro/cellular, or human‐based research); (iii) type of EDC exposure; (iv) melatonin intervention characteristics (dose, route of administration, timing relative to EDC exposure, and duration); (v) target tissues or organ systems assessed; (vi) primary outcome measures; and (vii) proposed mechanisms of action. Data extraction was conducted independently by two reviewers using a standardized, predefined data collection form adapted from previous reviews (Adepoju et al., 2025; Ajayi et al., 2025; Dukuze et al., 2026; Hakizimana et al., 2024; Kampire et al., 2025; Niyomugabo et al., 2026; Onohuean et al., 2022, 2025; Twambaze et al., 2025, 2026) to ensure consistency and minimize bias. Any discrepancies or disagreements in extracted data were resolved through joint discussion and consensus, and a third reviewer was consulted when necessary. Due to expected heterogeneity in study designs, models, and outcome measures, data were synthesized using a systematic approach, without any meta‐analysis.

### 2.5 Mechanistic categorization and thematic analysis

For this systematic review, all extracted data were organized and analyzed according to the underlying mechanisms by which melatonin mitigates EDC toxicity across multiple tissues. Each study was evaluated for its primary mechanistic pathway, and findings were grouped into thematic categories, including antioxidant and free radical scavenging activity, modulation of hormone receptors, regulation of gene and protein expression, stabilization of endocrine axes, mitochondrial and cellular protection, and epigenetic modulation. Studies were further categorized by target tissues or organ systems, such as reproductive, neuroendocrine, metabolic, immune, cardiovascular, and sensory systems, to assess multisystem interactions. A thematic analysis was conducted to identify recurring mechanistic patterns, correlations between tissue‐specific outcomes, and dose‐ or timing‐dependent effects of melatonin. This approach allowed for the identification of consistent protective pathways, areas with limited evidence, and gaps in the literature. Findings were synthesized systematically and in tabular form to provide a comprehensive, integrated overview of melatonin's mitigating effects against EDCs, highlighting the molecular, physiological, and functional mechanisms through which melatonin exerts protective actions across different biological systems.

### 2.6 Risk of bias assessment

To evaluate the methodological quality of the included studies, we conducted a systematic Risk of Bias (RoB) assessment tailored to study type. For animal studies, we applied the Systematic Review Center for Laboratory Animal Experimentation (SYRCLE)'s RoB tool, assessing domains such as randomization, blinding, and selective reporting. Human studies were evaluated using the Cochrane RoB 2.0 tool (for trials) or the Newcastle‐Ottawa Scale (NOS) (for observational), focusing on selection, comparability, and outcome measurement. In vitro studies were assessed using a modified Checklist for Reporting In‐vitro Studies (CRIS), examining experimental design, reproducibility, and statistical rigor. Two independent reviewers performed the assessments, with discrepancies resolved by consensus or a third reviewer. Studies were categorized as low, moderate, or high risk, and findings were summarized in tables for clarity (File S3). Sensitivity analyses were conducted to assess the influence of high‐bias studies on overall conclusions, ensuring robustness in our synthesis.

## 3 RESULTS

**FIGURE 1:**
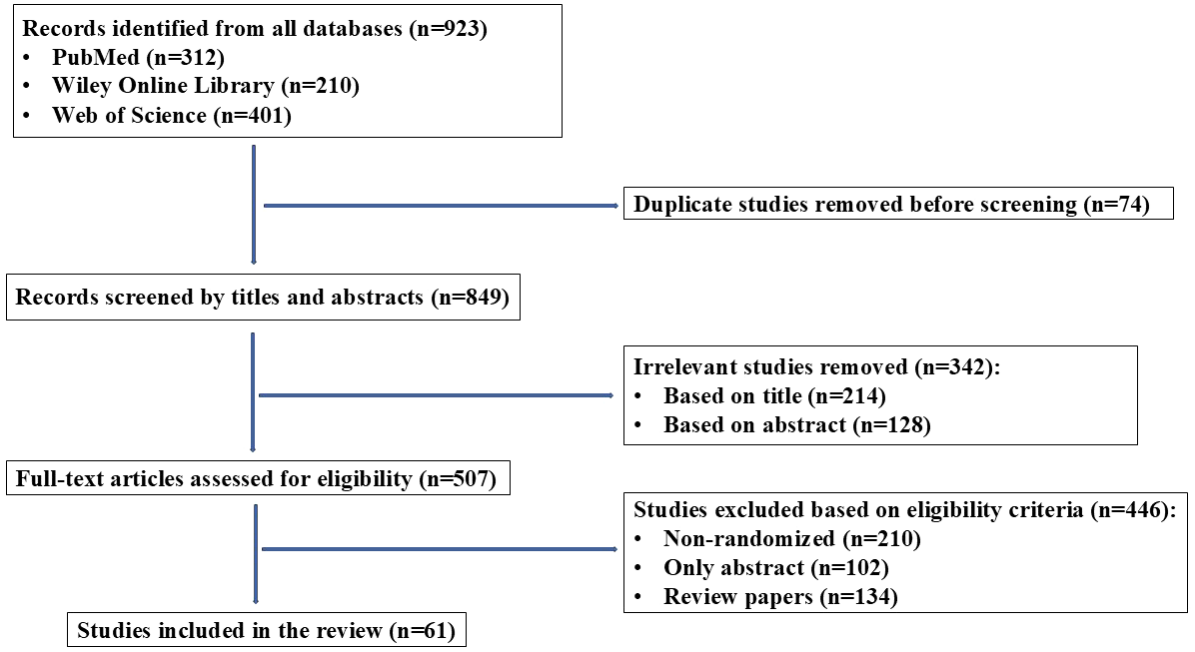
PRISMA flow chart of the article selection process.

Initially, a total of 923 articles were identified through systematic database searching, comprising 312 records from PubMed, 401 records from Web of Science, and 210 records from Wiley Online. After removing 74 duplicate records, 849 unique articles remained for further assessment. Title and abstract screening led to the exclusion of 342 articles that were clearly irrelevant to the topic, such as studies not involving endocrine disruptors, melatonin, or antagonistic/protective mechanisms. The remaining 507 articles underwent full‐text evaluation for eligibility. During this phase, 446 articles were excluded due to insufficient mechanistic data, lack of direct assessment of melatonin's antagonistic role, inappropriate study design, or absence of relevant biological endpoints. Consequently, 61 articles fully met the predefined inclusion criteria and were selected for qualitative synthesis in this review (Figure 1).

### 3.1 Antioxidant and free radical scavenging effects

The most extensively documented mechanism of melatonin's protection is its powerful capacity to act as both a direct free radical scavenger and an indirect enhancer of endogenous antioxidant systems. This fundamental property was demonstrated across 36 studies, revealing a consistent pattern of defense against the oxidative stress instigated by various EDCs (Table 2).

**TABLE 2:** List of studies linking melatonin antagonistic effects on endocrine disruption across multiple tissues.

Endocrine disruptors	Author and Reference	Physiological system	Animal model	Sample size	EDC exposure period and effects	Dose and period of melatonin treatment, and experimental setting (e.g., in vivo, in vitro, ex vivo)	Melatonin effects and mechanisms involved	Techniques for the assessment of parameters
Arsenic	Bali et al. (2016)	Hepatic	Male rat	24	15 days; liver dysfunction, oxidative stress	10 mg/kg/day, 15 days; in vivo	Improved liver enzymes; ↓oxidative stress	Biochemical assays, histopathology
Atrazine	Yang, Wang, et al. (2024)	Hepatic	Male rat	40	21 days; hepatic metabolic dysfunction	5 mg/kg/day, 21 days; in vivo	Alleviated metabolic dysfunction; regulated ER stress	Biochemical assays, RT‐PCR
B(a)P‐	Xu et al. (2021)	Female reproductive (ovary)	Female mouse	20/group	7 day; corpus luteum dysfunction, oxidative stress/apoptosis	15 mg/kg/day in vivo and 0.1 mM/L in vitro,7 days; in vivo	Reduced oxidative stress/apoptosis; preserved luteal function	Histopathology, Western blot, and biochemical assays
BDE‐209	Wang et al. (2024)	Female reproductive (ovary)	Female rat	NR	4 weeks; cuproptosis, disrupted estrous cycle	10 mg/kg/day, 60 days; in vivo	Reversed cuproptosis‐associated gene expression	RT‐PCR, biochemical assays
BPA	Zhang et al. (2017)	Female reproductive (oocytes)	Female mouse	NR	7 days; impaired spindle, ↑ROS, ↓fertilization	30 mg/kg/day, 7 days; in vivo	Improved spindle/meiosis; ↓ROS/apoptosis; Prevented mPTP opening	Immunofluorescence, biochemical assays
	Abdullah & Rashid (2020)	Hematologic (RBCs)	Human RBCs	100	12 h; ↑lipid peroxidation, ↓GSH	50, 100, 150, 200, and 250 μg/mL, in vitro	Reduced lipid peroxidation; restored antioxidants	Biochemical assays
	Ebrahimi et al. (2021)	Digestive (gastric cells)	Cell culture/MKN45/HGF/MSC cells	NR	24 h and 72 h; ↑ROS/MDA, ↓GSH, cytotoxicity, DNA damage	0.5, 5, 50, and 100 μg/mL; in vitro	Reduced ROS/MDA; restored GSH	Flow cytometry, biochemical assays
	Flora et al. (2004)	Multiple tissues	Male rat	42	35 days; oxidative stress, tissue damage	5 mg/kg/day, 35 days; in vivo	Partially restored redox balance	Biochemical assays
	Gładysz et al. (2023)	Thyroid	Animal thyroid/liver homogenates	21	24 h; ↑lipid peroxidation	5 mM, 24 h; ex vivo	Prevented oxidative damage	Biochemical assays
	Ishtiaq et al. (2021)	Nervous (brain)	Male rat	30	4 weeks; apoptosis (p53/PUMA/Drp‐1)	4 mg/kg/day, 16 days; in vivo	Normalized p53/PUMA/Drp‐1 axis; decreased ROS/TBARS; normalized apoptosis markers	Western blot, Immunohistochemistry, and biochemical assays
	Anjum et al. (2011)	Male reproductive (testis)	Male rat	NR	14 days; ↓mitochondrial enzymes, ↑LPO	10 mg/kg/day, 14 days; in vivo	Restored mitochondrial enzymes; ↓LPO	Biochemical assays, histopathology
	Olukole et al. (2018)	Male reproductive (prostate)	Male rat	40	14 days; ↑prostatic index, hormonal imbalance	10 mg/kg/day, 14 days; in vivo	Ameliorated hormonal imbalance	Biochemical assays, histopathology
	Olukole et al. (2019)	Endocrine (adrenal)	Male rat	28	14 weeks; ↑corticosterone/ACTH, adrenal damage	10 mg/kg/day, 14 weeks; in vivo	Restored adrenal structure/hormones	Histopathology, biochemical assays
	Wahby et al. (2017)	Hepatic/Renal	Male rat	50	70 days; liver/kidney toxicity, oxidative stress	10 mg/kg/day, 70 days; in vivo	Protected liver/kidney from oxidative damage	Biochemical assays, histopathology
	Wu et al. (2013)	Male reproductive (germ cells)	Male rat	NR	10 days; oxidative stress, DNA damage	4–10 mg/kg, 10 days; in vivo	Reduced oxidative stress/DNA damage	Comet assay, biochemical assays
	El‐Missiry et al. (2014)	Nervous (cerebrum)	Male rat	30	6 weeks; apoptosis (death receptor pathway)	10 mg/kg/day, 3 days per week for 6 weeks; in vivo	Modulated death receptor pathway proteins	Western blot, biochemical assays
	Kobroob et al. (2018)	Renal (kidney)	Male rat	30	4 weeks; oxidative stress, mitochondrial damage	10 mg/kg/day, 4 weeks; in vivo	Maintained mitochondrial membrane potential	Biochemical assays, histopathology
	Kadir et al. (2024)	Hypothalamic–pituitary axis	Male rat	42	120 days; disrupted GnRH/LH/FSH expression	10 mg/kg/day, 120 days; in vivo	Restored GnRH/LH/FSH expression	RT‐PCR, biochemical assays
	Akçay et al. (2020)	Hepatic	Male rat	42	60 days; ↑oxidative stress, adipocytokine imbalance	20 mg/kg/day, 60 days; in vivo	Reduced liver oxidative stress; normalized adipocytokines	Biochemical assays
	Yao et al. (2023)	Mice and human colonic epithelial cell (NCM460) models	Mice	N/R	Induced colonic DNA damage, oxidative stress and G2/M cell cycle arrest	In vivo (20 mg/kg/d) In vitro (10, 20, and 50 μM)	Ameliorated BPA effects by downregulating the expression of NOX family‐related genes, reversing the inhibition of the base excision repair pathway, promoting the activation of the non‐homologous end‐joining pathway, and suppressing the mRNA and protein expression of ATM, Chk1/2, and p53.	RT‐qPCR, Western Blotting, COMET assay, Immunofluorescence, Molecular docking
BPF/BPS	Turker Yavas et al. (2024)	Skeletal (bone)	Male rat	80	4 weeks; altered bone elasticity	Dose not specified, 4 weeks; in vivo	Improved bone mechanical properties	Mechanical testing, biochemical assays
BPS	Kumar et al. (2021)	Male Reproductive (testis)	Male hamster	NR	4 weeks; oxidative stress/inflammation	10 mg/kg/day, 4 weeks; in vivo	Modulated Nrf‐2/HO‐1 and SIRT‐1/FOXO‐1 pathways	RT‐PCR, biochemical assays
	Chouchene et al. (2025)	Female reproductive (ovary)	Male mouse	30	4 weeks; ovarian toxicity, dysregulated autophagy/mitophagy	10 mg/kg/day, 4 weeks; in vivo	Modulated autophagy/mitophagy pathway genes	RT‐PCR, biochemical assays
Cadmium	Lamtai et al. (2021)	Nervous (hippocampus)	Male rat	NR	4 weeks; cognitive deficits, oxidative stress	4 mg/kg/day, 60 days; in vivo	Alleviated cognitive deficits; ↓oxidative stress	Behavioral tests, biochemical assays
	Poliandri et al. (2006)	Endocrine	Male rat	NR	4 weeks; oxidative stress, altered gene expression	3 μg/mL 1 month; in vivo	Restored redox balance; normalized gene expression	RT‐PCR, biochemical assays
	Şensoy (2024)	Respiratory (lungs)	Male mouse	24	21 days; lung wall thickening, inflammation	3 mg/kg/day, 21 days; in vivo	Prevented lung histopathology	Histopathology, biochemical assays
	Yang et al. (2021)	Hepatic	Male mouse	30	4 weeks; liver inflammation, oxidative stress	10 mg/kg/day, 4 weeks; in vivo	Suppressed oxidative stress/inflammation/apoptosis	Histopathology, biochemical assays
	Jiménez‐Ortega et al. (2012)	Neuroendocrine (pituitary)	Male rat	30	1 month; disrupted circadian clock genes	3 μg/mL 1, 1 month; in vivo	Restored circadian clock genes (Clock, Bmal1)	RT‐PCR, biochemical assays
	Venditti et al. (2021a)	Male reproductive (testis)	Male rat	30	4 weeks; ↓DAAM1/PREP expression	10 mg/kg/day, 4 weeks; in vivo	Restored DAAM1/PREP expression	RT‐PCR, immunohistochemistry
	Venditti et al. (2021b)	Male reproductive (blood‐testis barrier)	Male rat	24	2 months; disrupted BTB proteins	3 mg/kg/day, 2 months; in vivo	Restored BTB protein expression	RT‐PCR, immunohistochemistry
	Liu et al. (2024)	Hepatic	Male mouse	30	4 weeks; liver inflammation, fibrosis	10 mg/kg/day, 4 weeks; in vivo	Remodeled gut microbiota; activated intestinal FXR	RT‐PCR, biochemical assays
Cannabis sativa	Alagbonsi and Olayaki (2017)	Male reproductive system	Male rat	54	Oral CS 2 mg/kg for 30 days; ↓ sperm parameters, ↓ TAC, ↑ ROS, altered ROS–TAC score indicating oxidative stress	Melatonin 4 mg/kg (alone or combined with vitamin C 1.25 g/kg), 30 days, in vivo	Combined melatonin + vitamin C synergistically restored redox balance, reduced ROS, improved TAC and sperm parameters	ROS measurement, Total Antioxidant Capacity (TAC), ROS–TAC score, sperm parameter analysis
	Alagbonsi and Olayaki (2016)	Male reproductive system	Male rat	55	20, 30, and 40 days ↓GnRH, ↓ LH, ↓testosterone but increased ↑estradiol and ↑prolactin.	Melatonin 4 mg/kg (alone or combined with vitamin C 1.25 g/kg) for 20, 30, or 40 days	↑GnRH, ↑LH, ↑testosterone but increased ↓estradiol and ↓prolactin when combined with Vitamin C.	Hormonal assay with ELISA
	Alagbonsi et al. (2016)	Male reproductive system	Male rat	55	20–40 days; ↓sperm count/motility/morphology, ↓ testicular/body weight ratio, ↓ total antioxidant capacity (TAC), ↑ lactate dehydrogenase (LDH)	Melatonin 4 mg/kg alone or combined with vitamin C 1.25 g/kg; 20–40 days, in vivo	Melatonin alone exacerbated CS‐induced testicular damage; combined melatonin + vitamin C ameliorated oxidative damage and improved sperm and histological parameters; mechanism: antioxidant modulation and redox balance restoration	Spectrophotometric TAC assay; Enzymatic colorimetric
	Abdulrahim et al. (2021)	CNS (brain)	Male rat	24	Oral CS 2 mg/kg for 14 days; ↑ G6PD, GPx, SOD; ↓ NO; no effect on total cholesterol, triacylglycerol, HDL‐C, MDA, CAT, GR, AChE	Melatonin 4 mg/kg; co‐administered with CS or alone; 14 days; in vivo	Melatonin enhanced G6PD, reduced NO, and normalized GPx and SOD activity altered by CS; mechanism: modulation of redox balance and antioxidant enzyme activity	Spectrophotometric/colorimetric enzyme assays, cholesterol/TG/HDL‐C enzymatic kits
	Oluwasola (2026)	Female reproductive	Male rat	50	14‐day oral administration of 2 mg/kg CS; decreased reproductive hormones	Melatonin 4 mg/kg, co‐administered orally for 14 days, in vivo	Restored levels of GnRH, FSH, LH, estradiol, progesterone, and prolactin by modulation of CB1R and CB2R signaling	ELISA, immunoenzymometric assays.
	Alagbonsi and Olaya (2018)	Male reproductive system	Male rat semen	5	In‐vitro exposure to THC (1 mM); reduced sperm progressive motility and kinematic parameters	Melatonin 5 mM, in‐vitro co‐treatment	Attenuated THC‐induced motility reduction, by modulation of CB1 and CB2 receptor signaling pathways	Computer‐assisted sperm analysis (CASA)
CPF	Karaoz et al. (2002)	Respiratory (lungs)	Male rat	NR	5 days; lung toxicity, oxidative stress	10 mg/kg/day, 6 days; in vivo	Reduced oxidative stress/lung injury	Histopathology, biochemical assays
DBP	Yang, Chen, et al. (2024)	Leydig cell	Male mouse	30	DBP induces ferroptosis of mouse Leydig cells via upregulating the expression of Sp2 and transcription of Vdac2 gene	10 mg/kg/day, 4 weeks; in vivo	Alleviates DBP‐induced ferroptosis of mouse Leydig cells via inhibiting oxidative stress‐triggered Sp2/VDAC2 signals.	HE staining, immunohistochemistry, Western blot, sequencing, and biochemical analysis.
DEHP	Liu et al. (2019)	Female reproductive (ovary)	Male mouse	NR	4 weeks; ↑Xdh expression, oxidative stress	10 mg/kg/day, 4 weeks; in vivo	Normalized Xdh expression	RT‐PCR, biochemical assays
	Bahrami et al. (2018)	Male reproductive (testis)	Male mouse	32	14 days; ↓sperm quality, altered hormones	10 mg/kg/day, 14 days; in vivo	Improved reproductive hormone levels	Biochemical assays, sperm analysis
Ketoconazole	Alagbonsi et al. (2020)	Thyroid	Female rat	50	Oral 25 mg/kg ketoconazole for 14 days; ↑ TSH, ↑ T3, ↑ T4, ↑ iodine concentration (thyroid dysfunction)	Melatonin 4 or 10 mg/kg; pre‐, co‐, or post‐treatment for 14 days; in vivo	Pre‐ or post‐treatment with melatonin normalized TSH, T3, T4, and iodine levels; mechanism: modulation of thyroid hormone synthesis and iodination process	ELISA (microplate immunoassay) for TSH, T3, T4; Colorimetric microplate iodine assay; Spectrophotometric
Lead	Zhao et al. (2023)	Male reproductive (testis)	Male mouse	30	4 weeks; ↓sperm count/motility, oxidative stress	10 mg/kg/day, 4 weeks; in vivo	Inhibited NF‐κB‐mediated oxidative stress	Biochemical assays, sperm analysis
	Omeiza et al. (2021)	Nervous	Male rat	20	4‐week; impaired spatial learning & memory, increased anxiety, oxidative stress (↑MDA, ↑IL‐1β, ↑TNF‐α), decreased SOD & AChE activity	Melatonin 10 mg/kg, 4 weeks, in vivo	Restored antioxidant enzyme activities, normalized MDA, cytokine, and AChE levels	Behavioral assay, Biochemical assays
MEHP	Zhang et al. (2018)	Female reproductive (oocytes)	Female porcine oocytes	100	24 h; ↓polar body extrusion, spindle disruption	1 mM, 24 h; in vitro	Restored meiosis; ↓oxidative stress/apoptosis	Immunofluorescence, biochemical assays
Mercury	Stacchiotti et al. (2006)	Renal (kidney tubules)	Male rat	NR	24 h; tubular stress, renal injury	5 mg/kg/day, 24 h; in vivo	Normalized iNOS/eNOS expression	RT‐PCR, biochemical assays
	Rao & Chhunchha (2010)	Thyroid	Male rat	50	60 days; thyroid oxidative stress	5 mg/kg/day, duration 60 days; in vivo	Reduced oxidative stress	Biochemical assays
Nanosilica	Zhang et al. (2019)	Immune (Macrophages)	Cell culture/RAW264.7 macrophages	100	24 h; ↑apoptosis, ↑TNF‐α/cytokines	1 mM, 24 h; in vitro	Enhanced autophagy; ↓apoptosis	Flow cytometry, biochemical assays
Nickel	Lamtai et al. (2020)	Nervous (hippocampus)	Male rat	NR	8 weeks; anxiety/depression, memory impairment	4 mg/kg/day, 8 weeks; in vivo	Improved behavior; ↓oxidative damage	Behavioral tests, biochemical assays
NP	Tabassum et al. (2017)	Male reproductive (testis)	Male rat	30	45 days; ↑TBARS, testis damage	10 mg/kg/day, 45 days; in vivo	Ameliorated oxidative stress; improved histology	Histopathology, biochemical assays
	Goktepe (2022)	Cardiovascular (heart)	Male rat	21	15daysmyocardial damage, oxidative stress	100 mg/kg/day, 15 days; in vivo	Reduced myocardial oxidative stress; improved histology	Histopathology, biochemical assays
Oxybenzone	Shi et al. (2025)	Female reproductive (oocytes)	Male mouse	120	4 weeks; meiotic defects, mitochondrial dysfunction	1 × 10^−7^ M1, 6–12 weeks; in vivo	Regulated mitochondrial dynamics/Ca^2+^ signaling genes	RT‐PCR, immunofluorescence
PAHs	Shaker & Al‐Wasiti (2023)	Endocrine (occupational)	Human worker	100	Chronic exposure; altered MT1A receptor	N/A; observational study	Linked MT1A receptor to protective phenotype	RT‐PCR, biochemical assays
PCB126	Xia et al. (2024)	Hepatic	Male mouse	32	2 weeks; oxidative stress, inflammation	10 mg/kg/day, 2 weeks; in vivo	Inhibited ROS‐dependent NETs formation	Biochemical assays, histopathology
PM2.5	Guohua et al. (2021)	Respiratory (lungs)	Male mouse	30	8 weeks; lung injury, ferroptosis	10 mg/kg/day, 8 weeks; in vivo	Activated Nrf2 pathway; upregulated HO‐1/GSH biosynthesis	RT‐PCR, biochemical assays
Polyethylene microplastics	Farag et al. (2024)	Endocrine (adrenal)	Male rat	42	35 days; adrenal cortical damage, oxidative stress	5 mg/kg/day, 35; in vivo	Preserved adrenal structure/steroidogenesis	Histopathology, biochemical assays
TBBPA	Sun et al. (2022)	Male reproductive (testis)	Male swine	30	4 weeks; apoptosis/necroptosis	5 mg/kg/day, 4 weeks; in vivo	Modulated PTEN/PI3K/AKT pathway	Western blot, biochemical assays
	Kadir et al. (2024)	Hypothalamic–pituitary axis	Male rat	18	4 weeks; disrupted GnRH/LH/FSH expression	10 mg/kg/day, 4 weeks; in vivo	Restored GnRH/LH/FSH expression	RT‐PCR, biochemical assays
	Lin et al. (2025)	Female reproductive (granulosa)	Female human granulosa cells	100	24 h; ↓FSHR/connexin 43 expression	2 mM, 24 h; in vitro	Restored FSHR/connexin 43 expression	RT‐PCR, immunofluorescence
TCDD	Sarihan et al. (2015)	Cardiovascular (heart)	Male rat	28	45 days; cardiac injury, oxidative stress	5 mg/kg/day, 45 days; in vivo	Improved cardiac function; ↓oxidative stress	Echocardiography, biochemical assays
	Gül et al. (2018)	Female reproductive (ovary)	Female rat	30	4 weeks; ↓follicles, ovarian ultrastructure damage	10 mg/kg/day, 4 weeks; in vivo	Preserved ovarian follicular reserve/ultrastructure	Histopathology, biochemical assays

In the renal and digestive systems, melatonin directly counters EDC‐induced damage. Kobroob et al. (2018) demonstrated that melatonin reversed BPA‐induced oxidative stress and mitochondrial damage in the kidneys of male rats. The nervous system also benefits significantly from this antioxidant activity. Ishtiaq et al. (2021) found that melatonin decreased ROS and TBARS in the male rat brain exposed to BPA while boosting antioxidant enzymes. This neuroprotection extended to behavioral outcomes, as Lamtai et al. (2020, 2021) demonstrated that melatonin alleviated cognitive and behavioral deficits by reducing hippocampal oxidative stress in male and female rats exposed to cadmium and nickel, respectively. Goktepe (2022) also showed that melatonin reduced oxidative and inflammatory markers in the frontal cortex and hippocampus of the NP‐exposed male rat brain. Furthermore, Wahby et al. (2017) found that melatonin protected the liver and kidney from BPA toxicity by reducing oxidative damage markers in male rats. Finally, Flora et al. (2004) showed that co‐administration of melatonin partially restored redox balance in lead‐exposed male rats across multiple tissues.

Melatonin's antioxidant action proves critical for protecting reproductive health. In the male reproductive system, melatonin ameliorated oxidative stress and improved testicular histology while restoring mitochondrial enzymes and reducing lipid peroxidation in the testes of BPA‐exposed mice (Wu et al., 2013) and NP‐exposed rats (Tabassum et al., 2017). Alagbonsi and Olayaki (2017, 2018) and Alagbonsi et al. (2016) found that melatonin and vitamin C exacerbate *Cannabis sativa*‐induced testicular damage when administered separately but ameliorate it when combined in male albino rats via restoration of redox homeostasis. Against phthalates, Bahrami et al. (2018) reported that melatonin reduced oxidative stress and inflammation, improving sperm quality in DEHP‐exposed male mice. Zhao et al. (2023) demonstrated that it inhibited NF‐κB‐mediated oxidative stress to protect against lead‐induced spermatogenic damage in male mice. In the female reproductive system, Xu et al. (2021) showed that melatonin reduced oxidative stress and apoptosis in the corpus luteum of B(a)P‐exposed female mice. Zhang et al. (2017, 2018) observed that melatonin decreased ROS levels and reduced oxidative stress to improve oocyte quality and restore meiosis in BPA‐exposed female mouse oocytes and MEHP‐exposed porcine oocytes, respectively. Additionally, Liu et al. (2019) demonstrated that melatonin reduced ovarian oxidative stress by normalizing Xdh gene overexpression in DEHP‐exposed mice. On the nervous system, results by Omeiza et al. (2021) demonstrated that melatonin reinstated antioxidant enzyme activities, normalized lead‐induced elevations in MDA and pro‐inflammatory cytokines, restored AChE activity, and mitigated neuronal damage in male Wistar rats, demonstrating its neuroprotective action via antioxidant and anti‐inflammatory mechanisms. Further study by Abdulrahim et al. (2021) revealed how melatonin modulates brain redox homeostasis in *C. sativa*‐treated female rats by enhancing glucose‐6‐phosphate dehydrogenase (G6PD) activity and suppressing nitric oxide (NO) production, while differentially regulating antioxidant enzymes (GPx and SOD).

The endocrine and cardiovascular systems are similarly shielded. Poliandri et al. (2006) showed that melatonin restored the redox balance in the hypothalamus and anterior pituitary glands disrupted by cadmium exposure in male rats. Gładysz et al. (2023) found that melatonin prevented oxidative damage induced by NH_4_SCN and NaF in thyroid tissue, and Rao and Chhunchha (2010) demonstrated its efficacy in reducing oxidative stress in the thyroid gland of mercury‐exposed male rats. Within the adrenal gland, Olukole et al. (2019) showed that melatonin reduced oxidative stress, restoring structure and hormones in BPA‐exposed male rats, and Farag et al. (2024) found it preserved adrenal structure in male rats exposed to microplastics. For the heart, Goktepe (2022) observed that melatonin reduced myocardial oxidative stress and improved cardiac histology in NP‐exposed male rats, and Sarihan et al. (2015) reported that it reduced oxidative stress and improved cardiac function in TCDD‐exposed male rats. Hepatic protection is consistently mediated through oxidative mechanisms. Xia et al. (2024) found that it inhibited ROS‐dependent NETs formation, reducing inflammation in the liver of PCB126‐exposed male mice. Bali et al. (2016) demonstrated that melatonin improved liver enzymes and reduced oxidative stress in arsenic‐exposed male rats, while Akçay et al. (2020) showed it reduced liver oxidative stress and normalized adipocytokines in BPA‐exposed male rats. Yang et al. (2021) found that melatonin suppressed oxidative stress, inflammation, and apoptosis in the livers of cadmium‐exposed male mice, and Yang, Wang, et al. (2024) demonstrated that it alleviated atrazine‐induced hepatic metabolic dysfunction in male rats by regulating ER stress, a pathway linked to oxidative imbalance. In the respiratory system, melatonin attenuates direct oxidative injury. Karaoz et al. (2002) found that melatonin reduced oxidative stress and lung injury in male rats exposed to chlorpyrifos‐ethyl. Şensoy (2024) showed that it prevented cadmium‐induced lung histopathology by countering oxidative damage in pregnant mice, and Guohua et al. (2021) demonstrated that it inhibited PM2.5‐induced lung ferroptosis by activating the Nrf2 antioxidant pathway in male mice. Its protective effect in the musculoskeletal system has also been shown by Turker Yavas et al. (2024), who found that melatonin improved bone mechanical properties against BPF/BPS in male rats via systemic antioxidant effects.

### 3.2 Regulation of gene and protein expression

Melatonin counteracts EDC‐induced toxicity by modulating the expression of critical genes and proteins involved in oxidative stress, circadian rhythms, cell adhesion, and apoptosis. This mechanism was documented across 18 studies involving the male and female reproductive, nervous, endocrine (pituitary, pineal), hepato‐digestive, and renal systems (Table 2). Venditti et al. (2021a) demonstrated in the male reproductive system (testis) of cadmium‐exposed male rats that melatonin restored the expression of the cytoskeletal proteins DAAM1 and PREP, essential for spermatogenesis. Jiménez‐Ortega et al. (2012) showed in the anterior pituitary of male rats that melatonin restored the disrupted 24‐h rhythmic expression of core circadian clock genes (Clock, Bmal1) induced by cadmium. In the female reproductive system (ovary), Wang et al. (2024) found that melatonin reversed cuproptosis‐associated gene expression induced by BDE‐209 in female rats. Further study by Poliandri et al. (2006) demonstrated in the endocrine system that melatonin normalized cadmium‐altered expression of redox‐related and clock genes in male rats. Guohua et al. (2021) demonstrated protection in the respiratory system, indicating that melatonin inhibited PM2.5‐induced lung ferroptosis via activation of the nuclear factor erythroid 2–related factor 2 (Nrf2) antioxidant pathway in male mice. Melatonin enhanced Nrf2 translocation into the nucleus, which upregulated downstream genes like HO‐1 and GSH biosynthesis enzymes. This boosted cellular defense systems, reduced glutathione depletion, and inhibited the accumulation of lethal lipid peroxides (e.g., 4‐HNE) in lung epithelial cells.

Liu et al. (2019) reported in the female reproductive system that melatonin normalized the overexpression of the Xdh gene, a key source of oxidative stress in DEHP‐exposed mouse ovaries. El‐Missiry et al. (2014) showed in the nervous system (cerebrum) of male rats that melatonin modulated the expression of death receptor pathway proteins to prevent BPA‐induced apoptosis. Furthermore, Kadir et al. (2024) found that melatonin restored BPA‐disrupted expression of the hypothalamic–pituitary‐gonadal (HPG) axis genes (GnRH, LH, and FSH) in male rats. In the digestive system (colon), melatonin reduced BPA‐induced DNA damage signaling by lowering NOX expression (Yao et al., 2023) in mice. In the hepatic system, melatonin inhibited ROS‐dependent NETs formation by modulating associated gene expression in PCB126‐exposed male mice (Xia et al., 2024). In the male reproductive system, melatonin suppressed the Sp2/voltage‐dependent anion channel 2 (VDAC2) axis to inhibit DBP‐induced ferroptosis in mouse Leydig cells (Yang, Chen, et al., 2024) and modulated the PTEN/PI3K/AKT pathway to antagonize TBBPA‐induced cell death (Liu et al., 2015). In the hepatic system, melatonin alleviated atrazine‐induced metabolic dysfunction in male rats by regulating ER stress response genes (Yang, Wang, et al., 2024). It was further demonstrated in the male reproductive system that melatonin modulated the Nrf‐2/HO‐1 and SIRT‐1/FOXO‐1 pathways at the gene expression level in male hamsters (Kumar et al., 2021). Chouchene et al. (2025) found that melatonin modulated autophagy and mitophagy pathway genes to protect against BPS ovarian toxicity in female mice. In the renal system, Stacchiotti et al. (2006) found that melatonin normalized the expression of iNOS/eNOS in mercury‐exposed kidneys of male rats. Venditti et al. (2021b) found that melatonin also restored the expression of blood‐testis barrier (BTB) proteins disrupted by cadmium in male rats. In oocytes, Shi et al. (2025) found that melatonin improved outcomes by regulating genes involved in mitochondrial dynamics and calcium signaling after oxybenzone exposure in female mice.

### 3.3 Mitochondrial and cellular protection

Melatonin provides critical defense at the subcellular level by preserving mitochondrial integrity and preventing specific forms of regulated cell death. This mechanism, which directly safeguards cellular viability and function, was documented in 12 studies. The protection was pivotal across the male and female reproductive (Chouchene et al., 2025; Shi et al., 2025; Sun et al., 2022; Wang et al., 2024; Xu et al., 2021; Yang, Wang, et al., 2024; Zhang et al., 2017, 2018), renal (Kobroob et al., 2018), nervous (Omeiza et al., 2021), immune (Zhang et al., 2019), and hepatic (Yang et al., 2021) systems (Table 2).

Protection by melatonin against di‐butyl phthalate (DBP) in the male reproductive system was shown to be mediated by downregulating the transcription factor Sp2, which in turn reduced the expression of the mitochondrial VDAC2 in male rats (Yang, Chen, et al., 2024). Melatonin also directly chelated excess copper and restored mitochondrial metabolism in female rats, preventing the toxic aggregation of lipoylated proteins in the tricarboxylic acid (TCA) cycle. This intervention halted this unique form of copper‐dependent mitochondrial cell death, protecting ovarian follicles (Wang et al., 2024). Another study showed that melatonin improved mitochondrial dynamics and calcium (Ca^2+^) signaling in oxybenzone‐exposed mouse oocytes (Shi et al., 2025). Also, Zhang et al. (2018) found that melatonin restored meiosis in porcine oocytes exposed to MEHP and directly scavenged MEHP‐generated ROS, preventing oxidative damage to the meiotic spindle apparatus. It also downregulated the expression of pro‐apoptotic proteins like Bax and cleaved caspase‐3 while upregulating the anti‐apoptotic protein Bcl‐2. This rebalancing of apoptotic regulators safeguarded oocyte health and allowed for normal polar body extrusion in female mice (Zhang et al., 2017).

Melatonin modulated the expression of mitochondrial fusion (Mfn2) and fission (Drp1) proteins to restore a healthy mitochondrial network. Concurrently, it stabilized endoplasmic reticulum (ER)‐mitochondria contacts and regulated the IP3R‐GRP75‐VDAC1 channel complex, preventing pathological Ca^2+^ release from the ER and subsequent mitochondrial calcium overload, which is a key trigger for apoptosis. Melatonin also prevented the opening of the mitochondrial permeability transition pore (mPTP) induced by BPA, thereby maintaining the mitochondrial membrane potential (MMP), inhibiting cytochrome C release into the cytoplasm, and blocking the activation of the caspase‐9/‐3 executioner cascade, and consequently, arresting the intrinsic apoptotic pathway in the mouse (Zhang et al., 2017). Xu et al. (2021) also demonstrated that melatonin inhibits the mitochondrial cytochrome C/caspase‐3 apoptotic pathway by preventing the initiation of the apoptosome complex caused by B(a)P, ensuring the survival and steroidogenic function of luteal cells in female mice. Zhang et al. (2019) showed protection in the immune system by decreasing apoptosis in nanosilica‐exposed macrophages (a mouse macrophage cell line, RAW264.7), and the key mechanism was the enhancement of cytoprotective autophagy, as melatonin activated the AMPK/ULK1 signaling pathway while inhibiting mTOR. This increased autophagic flux helped clear damaged organelles and protein aggregates, reducing cellular stress and inhibiting the subsequent activation of caspase‐3‐mediated apoptosis. A study by Ishtiaq et al. (2021) showed the anti‐apoptotic effect of melatonin against BPA insult in the male rat brain via normalization of the p53/PUMA/Drp‐1 axis and reducing the activation of Drp‐1, a protein responsible for pathological mitochondrial fission, thereby preserving mitochondrial network integrity and blocking the release of apoptotic factors.

Sun et al. (2022) showed melatonin's protection against TBBPA‐induced dysfunction in the male swine reproductive system. Melatonin allowed for the activation of PI3K and its downstream effector AKT. Phosphorylated AKT then inactivated pro‐apoptotic proteins (like Bad) and inhibited the necroptosis executor RIPK3, shifting the cellular balance away from both programmed death pathways. The study of Chouchene et al. (2025) showed that melatonin modulated autophagy and mitophagy pathways to defend against BPS‐induced ovarian toxicity in female mice. Melatonin activated the PINK1/Parkin‐mediated mitophagy pathway to selectively remove damaged mitochondria. Simultaneously, it fine‐tuned general autophagy via the AMPK/mTOR pathway, ensuring efficient clearance of toxic protein aggregates while preventing excessive self‐digestion, thus maintaining ovarian follicular health. Yang, Wang, et al. (2024) indicated protection in the hepatic system in male rats. This work demonstrated that melatonin alleviated atrazine‐induced hepatic metabolic dysfunction. The mechanism involved the regulation of the unfolded protein response (UPR) during ER stress. Melatonin attenuated the overactivation of the PERK‐eIF2α‐ATF4‐CHOP arm of the UPR. By reducing CHOP expression, a key mediator of ER stress‐induced apoptosis, melatonin mitigated downstream mitochondrial dysfunction and preserved hepatocyte survival and metabolic function. Kobroob et al. (2018) demonstrated mitochondrial protection against BPA‐induced toxicity in the renal system of male rats.

### 3.4 Endocrine axis stabilization

Melatonin demonstrates a pronounced ability to restore equilibrium within the body's critical hormonal axes, counteracting the systemic disruption caused by EDCs. This mechanism, focused on normalizing hormone levels, protecting endocrine organ structure, and maintaining feedback loops, was documented in 7 studies involving the hypothalamic–pituitary–adrenal (HPA) axis, the HPG axis, and the pineal gland (Table 2).

Olukole et al. (2018) demonstrated stabilization within the male HPG axis by showing that melatonin ameliorated BPA‐induced hormonal imbalance in the prostate, specifically reversing the decrease in testosterone and the increase in estrogen and prostate‐specific antigen (PSA) in male rats. Olukole et al. (2019) showed direct HPA axis stabilization in the adrenal gland, where melatonin reduced oxidative stress, restored adrenal cortical architecture, and normalized BPA‐elevated levels of corticosterone and adrenocorticotropic hormone (ACTH) in male rats. Melatonin also improved altered reproductive hormone levels in DEHP‐exposed male mice, which correlated with recovered sperm quality (Liu et al., 2025). Farag et al. (2024) found that melatonin stabilized the endocrine axis by counteracting PE‐MPs–induced HPA axis disruption, restoring serum cortisol levels, and suppressing the compensatory elevation of ACTH in male rats. Gül et al. (2018) provided evidence for female HPG axis support, demonstrating that melatonin preserved ovarian follicular reserve and ultrastructure after TCDD exposure, thereby maintaining the integrity of the ovarian endocrine unit in female rats. Kadir et al. (2024) directly addressed central HPG axis regulation at the transcriptional level, showing that melatonin restored the BPA‐disrupted expression of key hypothalamic–pituitary genes, including GnRH, LH, and FSH in male rats. Other teams also showed that melatonin exerted regulatory control over the hypothalamic–pituitary–thyroid (HPT) axis of rats by modulating thyroid hormone synthesis and iodination processes, thereby normalizing ketoconazole‐induced elevations in TSH, T3, T4, and iodine levels (Alagbonsi et al., 2020).

### 3.5 Hormonal receptor and signaling pathway modulation

Melatonin's protection involves direct interaction with cellular receptors and specific regulation of key intracellular signaling cascades. This targeted molecular mechanism was evidenced in 7 studies involving the endocrine, male reproductive, female reproductive, and other systems (Table 2). In the male reproductive system, Kumar et al. (2021) showed that melatonin acted by modulating the SIRT‐1/FOXO‐1 and Nrf‐2/HO‐1 pathways to reduce testicular oxidative stress and inflammation in BPS‐exposed male hamsters. Sun et al. (2022) demonstrated that melatonin antagonized TBBPA‐induced apoptosis and necroptosis in male swine testes by modulating the PTEN/PI3K/AKT cell survival signaling pathway. Yang, Chen, et al. (2024) showed that melatonin inhibited DBP‐induced ferroptosis in Leydig cells by suppressing the Sp2/VDAC2 signal in male rats. Zhao et al. (2023) reported that melatonin improved sperm parameters in lead‐exposed male mice by inhibiting NF‐κB‐mediated oxidative stress and inflammatory signaling. Chouchene et al. (2025) demonstrated in the female reproductive system that melatonin protected against BPS‐induced ovarian toxicity in female mice by modulating autophagy and mitophagy pathways, representing a targeted action on critical cellular degradation and quality control signaling cascades. Other authors also reported that melatonin mitigated *C. sativa*–induced suppression of reproductive hormones by modulating cannabinoid receptor (CB1R and CB2R) signaling in male (Alagbonsi & Olayaki, 2016) and female rats (Oluwasola, 2026). Also, by interacting with the receptor, melatonin improves sperm motility and kinematics in tetrahydrocannabinol (THC)‐treated rat sperm in vitro (Alagbonsi & Olayaki, 2018), just as vitamin C did (Alagbonsi & Olayaki, 2020).

### 3.6 Epigenetic modulation

Melatonin's protective repertoire extends to broader systemic and potential epigenetic mechanisms, indicating an ability to influence long‐term regulatory programs and inter‐organ communication. While this is an emerging area of focus within the context of EDC antagonism, 1 study provided evidence pointing toward these higher‐order effects. This action was observed in the mice hepatic system with systemic gut involvement (Table 2). It was revealed that melatonin's protection against cadmium‐induced liver inflammation and fibrosis was mediated through the remodeling of the gut microbiota and the subsequent activation of the intestinal Farnesoid X Receptor (FXR) signaling pathway. Activated intestinal FXR then sends endocrine signals via the gut‐liver axis that ultimately suppress hepatic bile acid synthesis (by downregulating CYP7A1) and inhibit inflammatory pathways (like NF‐κB) in the liver. This mechanism shows that melatonin acts not just on the target organ (liver) but also systemically by reprogramming the gut microbiome and its metabolic output (Liu et al., 2024).

### 3.7 Human and clinical evidence

While the overwhelming majority of evidence for melatonin's antagonistic effects against EDCs derives from animal models, only five of the 61 included studies involved human‐derived material or a human study population. These are consolidated in this section to clearly delineate the current state of human evidence, separate from the preclinical findings summarized above. This scarcity of clinical evidence, despite extensive and consistent preclinical support across animal models, represents a critical gap in the field and underscores the need for well‐designed human clinical trials to establish the translational relevance of melatonin as a protective agent against EDC‐induced toxicity.

Four studies used human‐derived cell lines or tissue rather than a human study population. In human gastric cancer cell lines, Ebrahimi et al. (2021) showed that melatonin reduced BPA‐induced ROS and MDA while restoring glutathione (GSH) levels. In human red blood cells exposed to BPA in vitro, Abdullah and Rashid (2020) reported that melatonin reduced lipid peroxidation and restored antioxidants. In human granulosa cells, Lin et al. (2025) found that melatonin restored the expression of FSHR and connexin 43 suppressed by BPA. Yao et al. (2023) used human colonic epithelial cell (NCM460) and reported the ameliorative effect of melatonin on BPA‐induced colonic DNA damage by scavenging NOX‐derived ROS, which further attenuates G2/M cell cycle arrest dependent on the ATM‐Chk1/2‐p53 axis. The only study involving an actual human study population identified in this review was an observational study by Shaker and Al‐Wasiti (2023), conducted among Iraqi refinery workers occupationally exposed to polycyclic aromatic hydrocarbons (PAHs). This study reported that a protective, anti‐cancer phenotype in PAH‐exposed workers was associated with melatonin and its MT1A receptor; engagement of this G‐protein‐coupled receptor can initiate signaling cascades leading to the phosphorylation and activation of various transcription factors and co‐regulators. Receptor‐mediated signaling was further suggested to influence the activity of DNA methyltransferases (DNMTs) and histone deacetylases (HDACs), pointing to a possible epigenetic mechanism (e.g., DNA methylation, histone acetylation) by which melatonin might contribute to genomic stability and a protective gene expression profile against carcinogenic EDCs. As this was an observational study without a randomized or interventional design, it provides correlative rather than causal evidence.

## 4 DISCUSSION

### 4.1 Summary of findings

This paper thoroughly illustrates how melatonin serves as a pleiotropic biological defense against toxicity caused by EDCs in a variety of physiological systems. Many EDCs, such as bisphenols, cadmium, phthalates, dioxins, and heavy metals, continuously interfere with redox homeostasis, mitochondrial integrity, hormonal signaling, circadian regulation, and tissue architecture, according to data from in vitro, in vivo, and a few human investigations. By reestablishing antioxidant capacity, regulating inflammatory pathways, restoring endocrine signaling, and maintaining cellular and structural integrity, melatonin intervention significantly reduces these detrimental effects. Melatonin protects the testicular microenvironment, enhances sperm parameters, and restores testosterone synthesis in the male reproductive system. Mechanistically, these effects are mediated by restoring important cytoskeletal and regulatory proteins, including DAAM1 and PREP (Venditti et al., 2021a), normalizing oxidative stress biomarkers, and suppressing NF‐κB‐driven inflammation (Zhao et al., 2023). The susceptibility of female reproductive tissues to EDCs was similar, especially regarding follicular development and oocyte quality. EDCs such as BPS, oxybenzone, and benzo(a)pyrene caused oxidative stress, dysregulated autophagy/mitophagy, meiotic abnormalities, and mitochondrial dysfunction, all of which compromised fertility (Chouchene et al., 2025; Shi et al., 2025; Xu et al., 2021). By reestablishing mitochondrial dynamics, lowering ROS, and restoring meiosis‐related signaling pathways, melatonin successfully mitigated these abnormalities. Remarkably, melatonin also restored the expression of connexin‐43 and the FSH receptor (FSHR) in human granulosa cells, demonstrating its translational significance to human ovarian physiology (Lin et al., 2025).

Melatonin had strong neuroprotective, metabolic, and immunomodulatory effects outside of reproduction. By suppressing oxidative stress, neuroinflammation, and apoptosis‐related pathways involving p53, PUMA, and Drp‐1, melatonin consistently improved cognitive, affective, and behavioral impairments in the nervous system caused by heavy metals and phenolic compounds (Omeiza et al., 2021). Melatonin restored redox balance, controlled ER stress responses, maintained mitochondrial function, and reversed biochemical indicators of organ damage in metabolic organs, including the liver and kidney (Kobroob et al., 2018; Yang, Wang, et al., 2024). Significantly, new research has shown that melatonin can alter gut microbiota and trigger intestinal FXR signaling to reduce hepatic fibrosis caused by cadmium, exposing a hitherto unrecognized gut–liver protective axis (Liu et al., 2024). All of these results point to melatonin as a system‐wide antagonist that can mitigate the complex toxicity of contemporary environmental EDCs.

### 4.2 Therapeutic potentials and translational implications

The therapeutic potentials discussed herein are drawn overwhelmingly from preclinical (animal and in vitro) findings (Sections 3.1, 3.2, 3.3, 3.4, 3.5, 3.6), with only a single human observational study identified in this review (Section 3.7); they should therefore be interpreted as hypotheses generated from preclinical mechanistic data rather than established clinical benefits, pending confirmation in human trials and with due consideration of the interspecies differences discussed in Section 4.4. Melatonin's promise as a low‐cost, multi‐target therapeutic adjuvant for reducing EDC‐induced pathophysiology is substantially supported by the body of research. It is especially well‐suited for complicated toxic exposures involving overlapping molecular processes because of its simultaneous ability to function as a direct free radical scavenger and an indirect regulator of antioxidant, inflammatory, apoptotic, and circadian gene networks.

It should be noted, however, that oxidative stress is not intrinsically pathological. At physiological, nanomolar concentrations, ROS such as hydrogen peroxide and the superoxide anion, generated via NADPH oxidases and the mitochondrial electron transport chain, act as second messengers that support redox signaling, metabolic regulation, and cellular adaptation to stress, a state now recognized as “oxidative eustress” as distinct from the tissue‐damaging “oxidative distress” produced by supraphysiological levels (Sies et al., 2017; Sies & Jones, 2020). Beyond signaling, ROS generated by phagocytic NADPH oxidase are essential for the respiratory burst that underlies innate immune defense against pathogens, and physiological ROS fluxes also regulate growth‐factor signaling, stem‐cell fate, and stress‐adaptive gene‐expression programs (Schieber & Chandel, 2014). Their complete elimination could therefore itself be disruptive to normal physiology. Thus, melatonin's therapeutic value in this context should be framed as restoring redox balance rather than abolishing oxidative stress altogether.

Based primarily on preclinical evidence, melatonin's capacity to maintain endocrine organ structure, preserve gamete quality, and restore reproductive hormone balance suggests potential uses in the treatment of infertility, especially in populations with high environmental exposure burdens, although this remains to be confirmed in human clinical trials. Preclinical findings on melatonin's ability to restore hormonal cycles and circadian gene expression (Clock, Bmal1) point to potential applicability to neuroendocrine illnesses and stress‐related dysregulation brought on by toxicants, though this too awaits testing in human populations. The single human observational study identified in this review, involving PAH‐exposed refinery workers with altered melatonin receptor signaling (Section 3.7), tentatively suggests that melatonin could be a protective intervention for populations exposed to the environment or at work, though this preliminary finding requires confirmation in larger clinical studies. Furthermore, its broad availability and generally favorable short‐term safety profile in human use (Section 4.5) make it a feasible candidate for future translational research, especially in low‐ and middle‐income countries where EDC exposure and healthcare constraints frequently overlap; however, this translational potential remains preliminary pending confirmatory human clinical trials.

### 4.3 Strengths over previous reviews

By providing a system‐level synthesis of melatonin's antagonistic activities against EDCs across the reproductive, endocrine, neurological, metabolic, immunological, cardiovascular, respiratory, and skeletal systems, this review contributes to the body of current literature. This paper incorporates new pathways like ferroptosis, cuproptosis, autophagy/mitophagy dysregulation, circadian clock disturbance, ER stress, gut microbiome remodeling, and receptor‐mediated signaling, in contrast to previous evaluations that mostly concentrated on oxidative stress. Furthermore, the research highlights melatonin's ability to effectively reverse EDC‐induced molecular and structural damage by emphasizing melatonin antagonism rather than just antioxidant supplementation. The originality of this synthesis is further reinforced by the inclusion of recent findings, which also represent the quickly developing understanding of melatonin as a master regulator of environmental stress resilience.

### 4.4 Interspecies differences in melatonin physiology: Implications for translation

Because most of the evidence in this review comes from rodent models, this section examines several biological differences between rodents and humans that matter for interpreting the findings. Humans sleep during their nighttime melatonin peak, while lab rats and mice are awake and active during that same peak, complicating how dosing timing translates between species. Genetic background also shapes melatonin production in ways that don't apply to humans: of 36 inbred mouse strains tested, only five retain the ability to make pineal melatonin (Goto et al., 1989), and C57BL/6 mice, one of the most widely used strains in toxicology research, carry a mutation that essentially abolishes their melatonin synthesis (Roseboom et al., 1998), meaning the results in this strain may reflect a pharmacological drug effect rather than restoring a natural deficit. Melatonin's effects on blood vessels also depend on which receptor subtype (MT1 causing constriction, MT2 causing dilation) and which vascular bed is involved, with both humans and rodents showing constriction in some vessels and dilation in others (Cook et al., 2011; Ozkalayci et al., 2021). So, a cardiovascular finding in one animal model doesn't reliably predict the human vascular response.

Also worthy of note is that some rodents, like hamsters, use melatonin as a seasonal breeding cue, and giving them melatonin can shut down testicular function the way it would signal “winter” in the wild, but this doesn't happen in standard lab rats, mice, or humans, none of which breed seasonally. So, studies using hamsters (Kumar et al., 2021) need extra caution in interpretation. Finally, most animal studies used doses far above what a human would take, producing blood melatonin levels well beyond the normal human range. Moreover, animals also clear melatonin from their system faster than humans do (Yeleswaram et al., 1997). So, the protective effects seen at those high and the fast‐cleared animal doses may not appear, or may behave differently, at the physiological doses used in people. Therefore, while the animal‐based evidence is strong and consistent, these differences call for caution when applying it to humans, reinforcing the need for human studies.

### 4.5 Limitations and future directions

There are still a number of limitations despite strong preclinical evidence. With little direct clinical evidence from humans, the majority of research is done in animal models or cell lines. Cross‐study comparisons are further complicated by variations in melatonin dosage, timing of administration, duration of exposure, and EDC combinations. Moreover, while real‐world exposure usually involves complex chemical combinations, much research concentrates on single toxicants. To determine the best dosage plans, therapeutic windows, and long‐term safety in exposed populations, future research should give top priority to carefully planned human observational and interventional studies. Additionally, sex‐specific reactions, developmental exposure windows, epigenetic control, and connections between endocrine signaling pathways and melatonin receptors (MT1/MT2) require more attention. Melatonin‐modulated system‐wide protective networks may be better understood by incorporating omics‐based techniques. Furthermore, existing human safety data for melatonin are derived overwhelmingly from short‐term trials. Systematic reviews of longer‐term (≥6 months) prospective studies have found a persistent scarcity of rigorously monitored safety data (Foley & Steel, 2019), and current guidance generally recommends short‐duration use pending further evidence (Colitta et al., 2026). Melatonin is also metabolized hepatically via cytochrome P450 1A2 and can interact with anticoagulants, antidiabetic agents, immunosuppressants, antihypertensives, and CYP1A2/CYP2C19 substrates such as fluvoxamine. These pharmacokinetic and pharmacodynamic interactions warrant caution and clinical supervision, particularly in populations with polypharmacy or chronic EDC exposure who may be considered for melatonin‐based interventions (Foley & Steel, 2019).

### 4.6 Conclusions

This review concludes with strong preclinical evidence, and some supportive but limited clinical evidence, that melatonin functions as a comprehensive biological antagonist against the harm caused by EDCs. In animal and in vitro models, melatonin provides multi‐layered protection across various physiological systems by maintaining redox equilibrium, endocrine signaling, circadian integrity, mitochondrial function, and tissue architecture. In the age of increasing ambient chemical exposure, melatonin is a promising candidate for future therapeutic and preventive use, owing to its wide mechanistic reach and favorable safety profile in short‐term human use. However, data on long‐term safety remain limited. Melatonin can interact with anticoagulants, antidiabetic agents, immunosuppressants, and other medications, and its translational potential remains to be established through well‐designed, long‐term human clinical trials.

## AUTHOR CONTRIBUTIONS

**Clenie Isingizwe:** Data curation; formal analysis; investigation; methodology; software; validation; visualization. **Gaudence Ndinganire:** Data curation; formal analysis; investigation; methodology; software; validation; visualization. **Abdullateef Isiaka Alagbonsi:** Conceptualization; data curation; formal analysis; investigation; methodology; project administration; software; supervision; validation; visualization.

## FUNDING INFORMATION

The authors have nothing to report.

## CONFLICT OF INTEREST STATEMENT

The authors declare that the research was conducted in the absence of any commercial or financial relationships that could be construed as a potential conflict of interest.

## ETHICS STATEMENT

The authors have nothing to report.

## CONSENT

The authors have nothing to report.

## Supporting information


**File S1.** PRISMA 2020 checklist.

**File S2.** Search strings used for each database.

**File S3.** Risk of bias assessment report.

## Data Availability

The data and the materials used in this review are available as Supplementary Files, including the PRISMA checklist for publication (File S1), the search strings (File S2), and the Risk of Bias Assessment Report (File S3). All these files can be found via the public FigShare DOI link: https://doi.org/10.6084/m9.figshare.32069334.
