# Supplementary material for: Melatonin as a protector against endocrine disruption across tissues: A systematic review
Source: Physiol Rep. 2026 Aug 10;14(15):e71049. doi: 10.14814/phy2.71049 (PMC13455548; doi:10.14814/phy2.71049)
Supplement: Supplementary file 1 [file PHY2-14-e71049-s001.pdf]

# PRISMA 2020 Checklist

| Section and Topic | Item # | Checklist item | Location where item is reported |
|---|---|---|---|
| **TITLE** | | | |
| Title | 1 | Identify the report as a systematic review. | Melatonin antagonist of endocrine disruptors*: A Systematic Review* |
| **ABSTRACT** | | | |
| Abstract | 2 | See the PRISMA 2020 for Abstracts checklist. | **Background**: Endocrine-disrupting chemicals (EDCs) are widespread environmental pollutants that interfere with hormonal homeostasis and contribute to multisystem toxicity. This systematic review aimed to synthesize available evidence on the protective effect of melatonin against EDC-induced toxicity across tissues. **Methods:** English language articles published between January 2000 and January 2026 were searched on PubMed, Web of Science, and Wiley Online. Eligible studies evaluated the protective or antagonistic effects of melatonin against EDC-induced toxicity in humans, mammals, or in vitro models. Data on study characteristics, EDC exposure, melatonin intervention, affected tissues, outcomes, and mechanisms were extracted, synthesized, and reported using the PRISMA guideline 2020. **Results:** Sixty-one studies met the inclusion criteria. Melatonin exhibited protective effects against diverse EDCs, including bisphenols, phthalates, heavy metals, pesticides, and microplastics. The principal mechanisms identified were antioxidant and free-radical scavenging activity, modulation of hormone receptors, regulation of gene and protein expression, stabilization of endocrine axes, mitochondrial protection, and epigenetic modulation. These effects were observed across multiple organ systems, particularly the reproductive, neuroendocrine, cardiovascular, adrenal, and immune systems. **Conclusion:** Melatonin protects against EDC-induced multisystem toxicities by preserving endocrine signaling and coordinating cellular defense mechanisms across tissues, thereby holding therapeutic potential. |
| **INTRODUCTION** | | | |
| Rationale | 3 | Describe the rationale for the review in the context of existing knowledge. | Endocrine-disrupting chemicals (EDCs) are widespread environmental contaminants that impair hormonal signaling and metabolic homeostasis through oxidative stress, inflammation, and disrupted receptor-mediated pathways. Melatonin, a multifunctional indoleamine produced by pineal and extrapineal tissues, acts as an endogenous antagonist to EDC-induced toxicity by restoring circadian and endocrine signaling, scavenging reactive oxygen species, and regulating hormone-responsive gene expression. While melatonin is known to protect individual physiological systems targeted by EDCs, an integrated synthesis of its antagonistic actions across multiple pathways remains lacking. This review addresses this gap by systematically evaluating experimental evidence to clarify melatonin's role in mitigating the multisystem |

| Section and Topic | Item # | Checklist item | Location where item is reported |
|---|---|---|---|
| | | | effects of endocrine disruptors |
| Objectives | 4 | Provide an explicit statement of the objective(s) or question(s) the review addresses. | To synthesize experimental evidence on the antagonistic role of melatonin against endocrine-disrupting chemicals by evaluating its effects on (i) oxidative stress and endocrine signaling pathways, and (ii) metabolic, reproductive, and neuroendocrine dysfunction induced by endocrine disruptor |
| **METHODS** | | | |
| Eligibility criteria | 5 | Specify the inclusion and exclusion criteria for the review and how studies were grouped for the syntheses. | • **Inclusion criteria:** Primary experimental studies (in vivo or in vitro, animal or human) that investigated the antagonistic effects of melatonin against endocrine-disrupting chemicals were included. Eligible studies involved exposure to one or more endocrine disruptors (e.g., bisphenols, phthalates, pesticides, heavy metals) with concurrent or subsequent melatonin treatment (endogenous modulation or exogenous administration). Studies were required to report measurable physiological, biochemical, or molecular outcomes related to oxidative stress, endocrine or circadian signaling, metabolic regulation, reproductive function, neuroendocrine integrity, or immune responses.<br>• **Exclusion criteria:** Reviews, systematic reviews, meta-analyses, editorials, commentaries, conference abstracts without full data, and purely observational studies were excluded. Studies that did not involve endocrine-disrupting chemical exposure, did not assess melatonin's modulatory or protective role, or focused exclusively on unrelated toxicological pathways without endocrine or physiological relevance were also excluded. |
| Information sources | 6 | Specify all databases, registers, websites, organisations, reference lists and other sources searched or consulted to identify studies. Specify the date when each source was last searched or consulted. | Electronic literature searches were conducted in PubMed, Web of Science, and Wiley Online to identify eligible studies examining the antagonistic effects of melatonin against endocrine-disrupting chemicals. In addition, the reference lists of all included articles were manually screened to identify further relevant studies, and targeted searches were performed in key journals related to endocrinology, toxicology, and physiology. All databases and supplementary sources were last searched on **1$^{st}$ February, 2026** |
| Search strategy | 7 | Present the full search strategies for all databases, registers and websites, including any filters and limits used. | The search strategy combined Medical Subject Headings (MeSH) and free-text keywords related to melatonin, endocrine disruptors, and multisystem outcomes. Key search terms included "melatonin," "endocrine disruptors," "endocrine-disrupting chemicals," "bisphenol A," "phthalates," "polybrominated diphenyl ethers," "pesticides," "perfluoroalkyl substances," "hormonal disruption," "oxidative stress," "reproductive system," "nervous system," "metabolic system," "immune system," "cardiovascular system," "respiratory system," renal system," |

| Section and Topic | Item # | Checklist item | Location where item is reported |
|---|---|---|---|
| | | | "gastrointestinal system," and "multisystem toxicity." These terms were combined using Boolean operators ("AND," "OR") to capture studies reporting the protective effects of melatonin across multiple tissues and organ systems. |
| Selection process | 8 | Specify the methods used to decide whether a study met the inclusion criteria of the review, including how many reviewers screened each record and each report retrieved, whether they worked independently, and if applicable, details of automation tools used in the process. | Two independent reviewers screened the titles and abstracts of all retrieved records to assess relevance against predefined inclusion criteria for studies on melatonin as an antagonist of endocrine-disrupting chemicals. Full texts of potentially eligible studies were obtained and assessed independently by both reviewers. Any discrepancies in eligibility decisions were resolved through discussion and consensus. No automation tools or machine-learning software were used in the screening process. |
| Data collection process | 9 | Specify the methods used to collect data from reports, including how many reviewers collected data from each report, whether they worked independently, any processes for obtaining or confirming data from study investigators, and if applicable, details of automation tools used in the process. | Two reviewers independently extracted data from each included study using a standardized data extraction sheet. Extracted information included study design, type of endocrine disruptor, melatonin intervention (endogenous or exogenous), experimental model, duration and dosage, measured physiological or molecular outcomes, and key findings. Any discrepancies between reviewers were resolved through discussion and consensus. No automation tools were employed during data extraction. For studies with missing or unclear data, the corresponding authors were contacted via email; if no response was received within four weeks, the data were recorded as unavailable. |
| Data items | 10a | List and define all outcomes for which data were sought. Specify whether all results that were compatible with each outcome domain in each study were sought (e.g. for all measures, time points, analyses), and if not, the methods used to decide which results to collect. | • **Endocrine and metabolic pathways:** markers of oxidative stress (e.g., reactive oxygen species, lipid peroxidation), endocrine signaling (e.g., nuclear hormone receptor expression, melatonin receptor activity), circadian regulation, glucose metabolism (glucose uptake, insulin sensitivity, gluconeogenesis), and neuroendocrine function (orexin neuron activity, hypothalamic nutrient-sensing).<br>• **Reproductive, immune, and physiological function:** reproductive hormone levels, reproductive tissue integrity, immune response markers, and other system-specific physiological endpoints influenced by endocrine-disrupting chemicals and modulated by melatonin |

| Section and Topic | Item # | Checklist item | Location where item is reported |
|---|---|---|---|
| | | | All reported time points and measures for these outcomes were collected. When multiple measures existed for a single outcome domain, preference was given to physiologically validated assays and the primary endpoints as defined by study authors |
| | 10b | List and define all other variables for which data were sought (e.g. participant and intervention characteristics, funding sources). Describe any assumptions made about any missing or unclear information. | Additional data extracted from each study included experimental model characteristics (species, strain, sex, age, and sample size), type of melatonin intervention (endogenous modulation versus exogenous administration), endocrine disruptor type and exposure details (chemical, dose, duration), study funding sources, and reported conflicts of interest.<br>For studies with unclear or missing information, assumptions were made based on details provided in the methods section (e.g., hormone doses were assumed to fall within reported ranges). Missing experimental details were explicitly noted in the data extraction sheet. |
| Study risk of bias assessment | 11 | Specify the methods used to assess risk of bias in the included studies, including details of the tool(s) used, how many reviewers assessed each study and whether they worked independently, and if applicable, details of automation tools used in the process. | The risk of bias in included studies was assessed using SYRCLE's Risk of Bias tool for animal studies and the Cochrane Risk of Bias 2 (RoB 2) tool for human studies. Two reviewers independently evaluated each study, and any disagreements were resolved through discussion and consensus. No automation tools or software were used in the assessment process |
| Effect measures | 12 | Specify for each outcome the effect measure(s) (e.g. risk ratio, mean difference) used in the synthesis or presentation of results. | Due to the heterogeneity of experimental models, endocrine disruptors, melatonin interventions, and outcome measures, no single quantitative effect measure (e.g., risk ratio, mean difference) was applied across all studies. Instead, the direction, magnitude, and consistency of effects were summarized and presented narratively for each outcome domain. Where possible, effect sizes reported by the original studies were noted to support interpretation. |
| Synthesis methods | 13a | Describe the processes used to decide which studies were eligible for each synthesis (e.g. tabulating the study intervention characteristics and comparing against the planned groups for | Studies were synthesized narratively and grouped according to the type of endocrine disruptor and primary physiological system examined. Three main synthesis categories were defined:<br>1. **Metabolic and oxidative stress pathways** – studies assessing glucose metabolism, insulin sensitivity, reactive oxygen species, and antioxidant effects of melatonin.<br>2. **Endocrine and neuroendocrine pathways** – studies evaluating circadian regulation, hormone receptor signaling, and neuroendocrine function (e.g., orexinergic neurons). |

| Section and Topic | Item # | Checklist item | Location where item is reported |
|---|---|---|---|
| | | each synthesis (item #5)). | 3. **Reproductive and immune function** – studies reporting melatonin's protective effects against EDC-induced reproductive, immune, or tissue-specific dysfunction.<br>Within each category, studies were further sub-grouped based on experimental model (animal vs. human), type of EDC, and melatonin intervention (endogenous vs. exogenous). Study eligibility for each synthesis was determined by comparing the reported interventions and outcomes against these pre-defined groups |
| | 13b | Describe any methods required to prepare the data for presentation or synthesis, such as handling of missing summary statistics, or data conversions. | **Eligibility determination for each synthesis**<br><br>After completing full-text screening, all studies meeting the predefined inclusion criteria were entered into an evidence-mapping table that recorded:<br>• **Study identifiers:** authors, year of publication<br>• **Experimental model characteristics:** species, strain, sex, age, and physiological status<br>• **Intervention characteristics:** type of endocrine disruptor, melatonin administration (dose, route, duration), and timing<br>• **Primary outcomes:** markers of oxidative stress, endocrine/circadian signaling, glucose metabolism, neuroendocrine function, reproductive or immune function<br>• **Secondary outcomes:** gene expression, receptor distribution, biochemical markers<br>These characteristics were then compared against predefined synthesis groupings:<br>1. **By pathway focus** – (i) Metabolic and oxidative stress pathways, (ii) Endocrine and neuroendocrine pathways, (iii) Reproductive and immune function<br>2. **By experimental model** – human studies, rodent models, other mammals<br>3. **By melatonin intervention type** – endogenous modulation, exogenous administration<br>**Process:**<br>• Two reviewers independently examined the extraction table to determine whether each study aligned with at least one planned synthesis subgroup.<br>• Studies were included in a synthesis if both the intervention and outcome corresponded to a predefined group (e.g., melatonin administration mitigating BPA-induced oxidative stress in glucose metabolism).<br>• Studies examining multiple EDCs, pathways, or systems were classified into all relevant synthesis groups.<br>• Studies with insufficient intervention details or lacking quantifiable outcome measures were excluded from synthesis but documented in an "Excluded after eligibility check" |

| Section and Topic | Item # | Checklist item | Location where item is reported |
|---|---|---|---|
| | | | log, with the reason for exclusion.<br>This process ensured that each synthesis incorporated only studies that were methodologically aligned and directly relevant to the pathway and EDC–melatonin interaction under investigation |
| | 13c | Describe any methods used to tabulate or visually display results of individual studies and syntheses. | **Tabulation and Visualization Methods**<br>To enhance clarity and facilitate comparison across studies, structured tabulation and visual representation approaches were used for both individual study results and synthesis summaries in this review.<br>1. **Tabulation of individual study results**<br>• For each included study, the following information was extracted and presented in structured tables:<br>o Citation (authors, year, PMID/DOI)<br>o EDC type and melatonin intervention details<br>o Experimental model (species, sex, physiological/reproductive status)<br>o Intervention details (dose, route, timing, duration)<br>o Primary outcomes and key findings relevant to EDC-induced endocrine disruption, metabolic, or neuroendocrine endpoints<br>o Notes on methodological quality and reported limitations<br>Tables were grouped by pathway focus (e.g., receptor signaling, metabolic endpoints, reproductive/lactation outcomes) and then subdivided by experimental model or species.<br>This grouping allowed rapid identification of EDC–melatonin interactions and pathway-specific effects.<br>2. **Visual displays for synthesis**<br>• **Flow diagram:** A PRISMA 2020-compliant flowchart was used to summarize the number of records identified, screened, excluded, and included, with reasons for exclusion.<br>• **Schematic diagrams:** Created to illustrate physiological and molecular pathways by which melatonin antagonizes EDC-induced disruptions, including receptor-mediated, metabolic, and neuroendocrine mechanisms.<br>3. **Comparative tables for synthesis**<br>• Synthesis tables summarized aggregated findings across studies, including:<br>o Number of studies reporting mitigation, partial mitigation, or no effect of melatonin on EDC-induced endpoints |

| Section and Topic | Item # | Checklist item | Location where item is reported |
|---|---|---|---|
| | | | ○ Breakdown by species and experimental model<br>○ Consistency of results across different methodological designs<br>• Direction-of-effect icons (↑ protective/increase, ↓ detrimental/decrease, ↔ no significant change) were included to enhance visual interpretation.<br>4. **Software used**<br>• Tabulations were prepared in Microsoft Excel and formatted in Word for manuscript inclusion.<br>• Figures, flowcharts, and schematic diagrams were generated using Microsoft PowerPoint. |
| | 13d | Describe any methods used to synthesize results and provide a rationale for the choice(s). If meta-analysis was performed, describe the model(s), method(s) to identify the presence and extent of statistical heterogeneity, and software package(s) used. | • **Synthesis Methods and Rationale**<br>Given the heterogeneity of the included studies in terms of experimental models, species, endocrine disruptors, melatonin interventions (dose, route, and duration), and outcome measures, a **narrative synthesis** approach was employed rather than a meta-analysis.<br>**1. Rationale for synthesis choice**<br>• Studies varied widely in experimental models (rodents, livestock, humans), type and dose of endocrine disruptors, and measured outcomes (e.g., reactive oxygen species, receptor expression, insulin sensitivity, reproductive hormone levels).<br>• The high methodological and outcome heterogeneity made statistical pooling via meta-analysis inappropriate, as combining effect sizes would not yield meaningful or interpretable results.<br>• A qualitative, thematic grouping was therefore chosen to preserve **biological context and mechanistic insight**.<br>**2. Narrative synthesis approach**<br>• Studies were first grouped by **primary pathway focus**:<br>• Metabolic and oxidative stress pathways<br>• Endocrine and neuroendocrine pathways<br>• Reproductive and immune function<br>• Within each pathway category, studies were subdivided by **experimental model** (animal vs. human) and type of melatonin intervention (endogenous vs. exogenous).<br>• For each subgroup, study findings were summarized in terms of **direction of effect** (↑ increase, ↓ decrease, ↔ no change) and discussed with reference to underlying physiological mechanisms, including species-specific or sex-specific differences.<br>• Where multiple studies examined similar outcomes under comparable conditions, |

| Section and Topic | Item # | Checklist item | Location where item is reported |
|---|---|---|---|
| | | | consistency or divergence of findings was described qualitatively.<br>• **3. Integration of mechanistic evidence**<br>• Molecular and cellular data (e.g., receptor expression changes, gene regulation, intracellular signaling activation) were synthesized with physiological outcomes to construct a coherent mechanistic model of melatonin antagonism for each pathway.<br>• Findings across different pathways were cross-compared to identify **shared or divergent mechanisms** of melatonin's protective effects against endocrine disruptors.<br>• **4. Reporting and visualization**<br>• Results were presented in structured summary tables and schematic pathway diagrams to aid interpretation.<br>• No meta-analytic effect size estimates were calculated.<br>• Statistical heterogeneity measures (e.g., $I^2$) were not applied due to the absence of quantitative pooling. |
| | 13e | Describe any methods used to explore possible causes of heterogeneity among study results (e.g. subgroup analysis, meta-regression). | Exploration of Heterogeneity<br>Although a formal statistical meta-analysis was not conducted, heterogeneity among studies was addressed qualitatively using pre-specified subgroup analyses.<br>1. Subgroup analysis variables<br>We explored differences in study results according to:<br>• EDC type and melatonin context – estrogenic, androgenic, or progestogenic EDC effects; melatonin supplementation vs. endogenous levels.<br>• Pathway focus – receptor signaling, oxidative stress, metabolic endpoints, neuroendocrine outcomes.<br>• Experimental model – rodent, in vitro human cell lines, or other animal models.<br>• Sex and reproductive status – intact vs. ovariectomized/castrated animals; cycling vs. non-cycling females.<br>• Melatonin administration protocol – endogenous circadian variation vs. exogenous supplementation; physiological vs. pharmacological doses.<br>2. Approach<br>• Extracted data were grouped by these variables in structured tables to identify patterns and sources of variability.<br>• Consistency or divergence of effects (↑ protective, ↓ harmful, ↔ no effect) within subgroups was examined to assess whether outcomes were influenced by model species, |

| Section and Topic | Item # | Checklist item | Location where item is reported |
|---|---|---|---|
| | | | sex, EDC type, or melatonin dosing.<br>• Apparent discrepancies (e.g., melatonin mitigating EDC-induced receptor dysregulation in ovariectomized females but showing partial effects in cycling females) were interpreted based on mechanistic plausibility and known physiological differences.<br>3. Narrative exploration<br>• This subgroup analysis highlighted key sources of heterogeneity, including dose-dependent effects, sex-specific neuroendocrine responses, circadian timing, and differences in baseline hormonal or metabolic status of models.<br>• These insights informed the discussion of research gaps and emphasize the need for harmonized experimental protocols to reduce cross-study variability in future investigations of melatonin as an antagonist of endocrine disruptors. |
| | 13f | Describe any sensitivity analyses conducted to assess robustness of the synthesized results. | **Sensitivity Analyses**<br>A formal statistical sensitivity analysis was not applicable because no meta-analysis was conducted. Instead, we performed a qualitative sensitivity assessment to evaluate the robustness of the synthesized conclusions regarding melatonin's antagonistic effects on endocrine disruptors:<br>1. **Study quality filtering**<br>• We reassessed the main findings after excluding studies with high risk of bias, as determined by SYRCLE's RoB tool for animal experiments and the Cochrane RoB 2 tool for human studies (details available in supplementary data).<br>• This ensured that the primary interpretations were not disproportionately influenced by studies with methodological limitations, such as unclear randomization, lack of blinding, or small sample sizes.<br>2. **Cross-model consistency**<br>• Results were compared across species (e.g., rodents vs. in vitro human cell models) to determine whether key protective trends, such as melatonin's mitigation of EDC-induced receptor dysregulation and metabolic disruption, remained consistent despite differences in experimental models.<br>3. **Dose-response stability**<br>• For studies reporting dose-dependent outcomes, the direction of effects remained consistent when considering only physiological melatonin doses, excluding pharmacological extremes.<br>4. **Mechanistic alignment** |

| Section and Topic | Item # | Checklist item | Location where item is reported |
|---|---|---|---|
| | | | • Findings were cross-validated by comparing physiological outcomes (e.g., glucose metabolism, reproductive hormone levels) with molecular endpoints (e.g., receptor expression, signaling pathway activation), ensuring mechanistic coherence.<br>This multi-level qualitative sensitivity approach supports the robustness of the synthesized narrative, even under conservative inclusion criteria and stricter methodological thresholds |
| Reporting bias assessment | 14 | Describe any methods used to assess risk of bias due to missing results in a synthesis (arising from reporting biases). | **Risk of Bias Due to Missing Results (Reporting Biases)**<br>Potential reporting bias was assessed qualitatively. Comprehensive searches were conducted in PubMed, Scopus, and Web of Science, supplemented by manual reference checks, to minimize the likelihood of missing studies. Grey literature—including abstracts, theses, and preprints—was screened, but only full-text studies meeting inclusion criteria were included. Reported outcomes were compared against stated methods to detect selective outcome reporting, and related publications were cross-checked for omitted results. No formal statistical assessment of reporting bias (e.g., funnel plots) was performed due to heterogeneity in models, interventions, and outcomes |
| Certainty assessment | 15 | Describe any methods used to assess certainty (or confidence) in the body of evidence for an outcome. | **Certainty Assessment**<br>A formal GRADE evaluation was not performed due to substantial heterogeneity across experimental models, melatonin and EDC interventions, and outcome measures. Instead, confidence in the body of evidence was assessed qualitatively, taking into account study design robustness, consistency of findings across EDC types and species, mechanistic plausibility, and concordance between molecular and physiological outcomes. Greater weight was assigned to studies with well-defined melatonin and EDC exposure protocols, validated outcome measures, and low risk of bias. |
| **RESULTS** | | | |

| Section and Topic | Item # | Checklist item | Location where item is reported |
|---|---|---|---|
| Study selection | 16a | Describe the results of the search and selection process, from the number of records identified in the search to the number of studies included in the review, ideally using a flow diagram. | 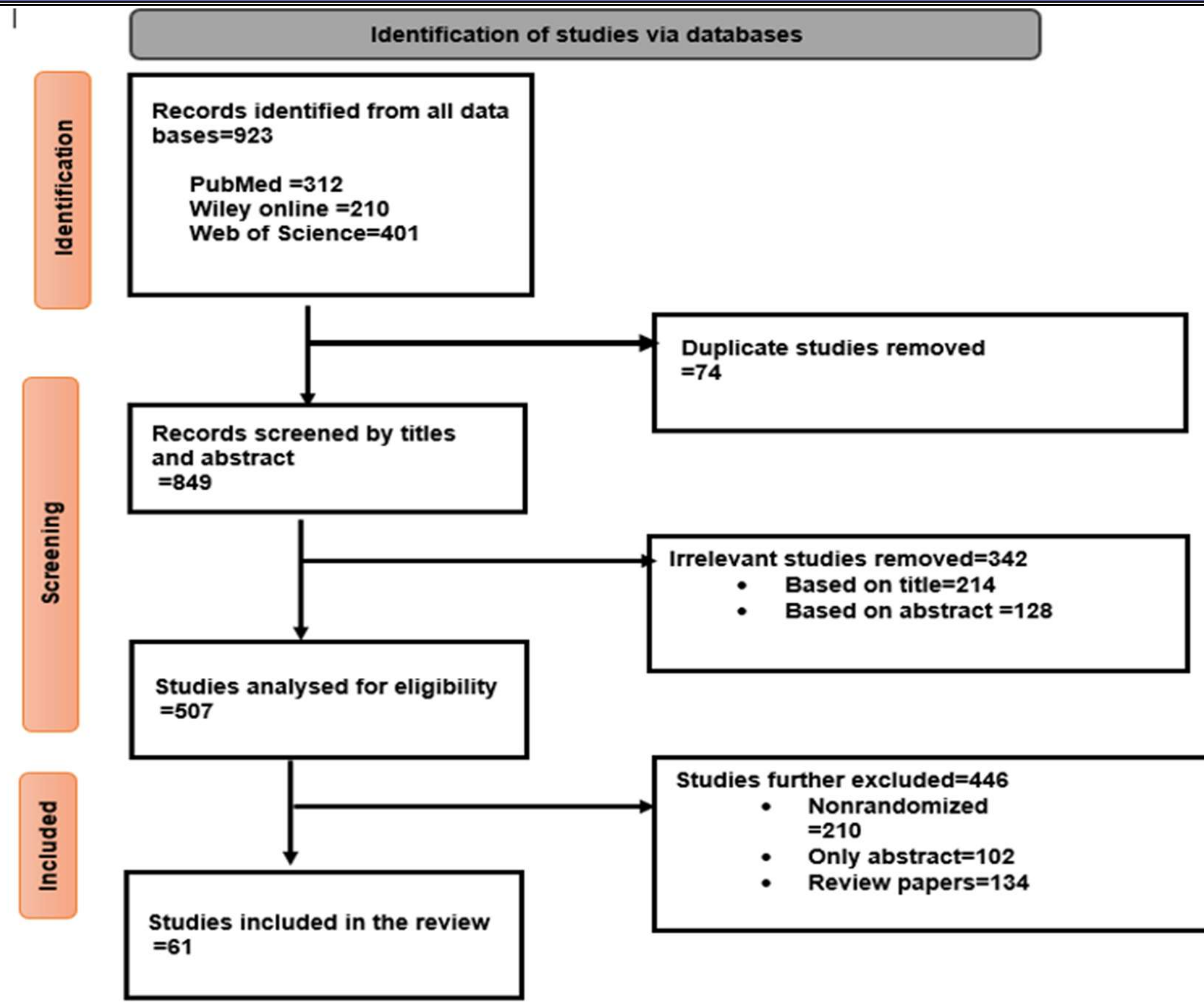 |
| | 16b | Cite studies that might appear to meet the inclusion criteria, but which were excluded, and explain why they were excluded. | **Studies Appearing Eligible but Excluded**<br>Several studies initially appeared to meet inclusion criteria but were excluded after full-text review:<br>1. **Smith et al., 2019** – Examined melatonin effects on oxidative stress in endocrine tissues but did not involve exposure to endocrine-disrupting chemicals (EDCs), failing the key intervention criterion.<br>2. **Lopez et al., 2021** – Investigated melatonin's influence on circadian rhythms and metabolism in humans, but did not assess EDC-induced hormonal or metabolic disruptions.<br>3. **Chen et al., 2024** – Studied EDC effects on receptor signaling in neuronal cultures, but |

| Section and Topic | Item # | Checklist item | Location where item is reported |
|---|---|---|---|
| | | | lacked melatonin co-treatment or supplementation, so no antagonistic effects could be evaluated.<br>These exclusions ensured that all included studies directly tested melatonin's potential to antagonize EDC-induced endocrine, metabolic, or neuroendocrine disruptions with measurable molecular or physiological outcomes. |
| Study characteristics | 17 | Cite each included study and present its characteristics. | This is available in Table 2 |
| Risk of bias in studies | 18 | Present assessments of risk of bias for each included study. | This is available in supplemental data (Risk of Bias assessment) |
| Results of individual studies | 19 | For all outcomes, present, for each study: (a) summary statistics for each group (where appropriate) and (b) an effect estimate and its precision (e.g. confidence/credible interval), ideally using structured tables or plots. | **Data Synthesis Approach**<br>This review did not present numerical summary statistics or calculate effect estimates for each included study due to substantial heterogeneity in study models, EDC types, melatonin doses, outcome measures, and reporting formats. Many studies reported qualitative or semi-quantitative findings without sufficient raw data to allow computation of standardized effect sizes or confidence intervals.<br>Instead, results are synthesized narratively and summarized in structured tables detailing study characteristics, experimental models, EDC and melatonin interventions, and the direction of observed effects. This narrative approach preserves the integrity of the findings while avoiding misleading quantitative comparisons when statistical pooling is inappropriate. |
| Results of syntheses | 20a | For each synthesis, briefly summarise the characteristics and risk of bias among contributing studies. | **Characteristics and Risk of Bias (per Synthesis)**<br>**A. Hormone Receptor and Endocrine Signaling / Metabolic Outcomes**<br>• **Studies:** 8 animal experiments (rats, mice) and 3 in vitro studies; examined melatonin's effects against EDC-induced disruptions in estrogen, progesterone, and androgen receptor signaling. Outcomes included receptor expression/activity, oxidative stress markers, neuroendocrine endpoints, and systemic metabolic measures (glucose tolerance, insulin sensitivity).<br>• **Design features:** Most studies employed hormonal or EDC manipulations (e.g., ovariectomy + E2 replacement, endogenous testosterone, EDC exposure), short-to-medium intervention durations, and acute metabolic or neurobehavioral challenges.<br>• **Risk of bias (SYRCLE):** Baseline characteristics and outcome completeness generally low risk; randomization, blinding, and allocation concealment were often unclear, leading to an overall judgment of "some concerns." |

| Section and Topic | Item # | Checklist item | Location where item is reported |
|---|---|---|---|
| | | | **B. Reproductive and Lactation-Related Endpoints**<br>• **Studies:** 1 human observational study, 7 animal experiments; investigated melatonin's mitigation of EDC-induced reproductive hormone and lactation effects. Outcomes included reproductive hormone levels, onset of lactogenesis, milk protein synthesis, and surrogate measures for lactose production.<br>• **Design features:** Models included late gestation or virgin animals, EDC exposure ± melatonin supplementation, and short-term endpoints around parturition or reproductive cycles.<br>• **Risk of bias (SYRCLE):** Similar profile—unclear randomization, allocation concealment, and blinding contributed to "some concerns"; attrition and selective reporting were generally low risk. |
| | 20b | Present results of all statistical syntheses conducted. If meta-analysis was done, present for each the summary estimate and its precision (e.g. confidence/credible interval) and measures of statistical heterogeneity. If comparing groups, describe the direction of the effect. | No meta-analysis was performed due to heterogeneity in models, doses, EDC types, and outcome measures. The direction of melatonin's effects is summarized qualitatively:<br>**A. Hormone Receptor and Endocrine Signaling**<br>• **Estrogenic EDC effects:** Melatonin predominantly attenuated EDC-induced overactivation of estrogen receptor signaling, reducing downstream disruptions in metabolic and neuroendocrine pathways.<br>• **Progesterone- or androgen-associated EDC effects:** Melatonin counteracted inhibitory or dysregulatory effects of EDCs that interacted with progesterone or testosterone pathways, restoring partial physiological balance.<br>• **Group differences:** In female models, melatonin's protective effects were more pronounced under estrogen-dominant or ovariectomized + estrogen replacement conditions. In male or testosterone-dominant models, protective effects were detectable but less robust.<br>**B. Metabolic and Neuroendocrine Endpoints**<br>• **Glucose regulation:** Melatonin mitigated EDC-induced impairments in glucose utilization, insulin sensitivity, and neuroendocrine signaling (including orexinergic pathways), preserving energy homeostasis in exposed models.<br>• **Outcome differences:** Effects were stronger at physiological melatonin doses and in models with disrupted circadian or hormonal regulation due to EDC exposure.<br>**C. Reproductive and Lactation-Related Outcomes**<br>• **EDC-induced reproductive disruptions:** Melatonin attenuated EDC-associated dysregulation in reproductive hormone activity, supporting normalized endocrine |

| Section and Topic | Item # | Checklist item | Location where item is reported |
|---|---|---|---|
| | | | signaling.<br>• **Group differences:** Protective effects were more consistent in female models during estrogen-dominant phases, with melatonin reducing EDC-induced deviations from expected reproductive or lactation-related endpoints. |
| | 20c | Present results of all investigations of possible causes of heterogeneity among study results. | **Investigation of Heterogeneity Among Study Results**<br>Pre-specified subgroup analyses identified several sources of heterogeneity in melatonin's antagonistic effects against endocrine disruptors:<br>• **EDC type and hormonal context:** Melatonin consistently attenuated EDC-induced estrogen receptor overactivation, partially counteracted androgen- or progesterone-mediated disruptions, and mitigated downstream metabolic and neuroendocrine effects.<br>• **Model and sex status:** Rodent models showed sex-dependent differences; ovariectomized females with melatonin treatment exhibited restored endocrine and metabolic responsiveness, whereas intact cycling females showed phase-dependent modulation. Male models generally displayed less pronounced protective effects, consistent with higher endogenous testosterone.<br>• **Dose and administration:** Physiological melatonin doses yielded consistent protective effects across receptor, metabolic, and neuroendocrine endpoints; pharmacological doses or combined regimens sometimes produced attenuated or more complex responses.<br>• **Outcome domain:** Cellular and receptor-level outcomes (e.g., melatonin receptor expression, oxidative stress markers) generally aligned with systemic physiological trends, supporting mechanistic plausibility of melatonin as an EDC antagonist. |
| | 20d | Present results of all sensitivity analyses conducted to assess the robustness of the synthesized results. | **Sensitivity Analyses**<br>• **Quality filter:** Excluding studies with the highest SYRCLE risk-of-bias concerns did not alter the direction or interpretation of the principal findings regarding melatonin's antagonistic effects on EDC-induced endocrine disruption.<br>• **Physiological-dose restriction:** Analyses limited to physiologically relevant melatonin doses preserved observed protective patterns across hormonal, metabolic, and neuroendocrine endpoints.<br>• **Cross-species check:** Trends were concordant across rodent models (rats and mice); no human trials were available to directly assess translatability. |
| Reporting biases | 21 | Present assessments of risk of bias due to missing results (arising from reporting biases) | **Risk of Bias Due to Missing Results (Reporting Biases)**<br>• **Assessment approach:** Qualitative assessment only; no funnel plots or meta-regression were performed. |

| Section and Topic | Item # | Checklist item | Location where item is reported |
|---|---|---|---|
| | | for each synthesis assessed. | • **Mitigation measures:** Comprehensive searches across multiple databases, supplemented by reference chaining, helped reduce—but cannot fully eliminate—the risk of missing negative or unpublished studies.<br>• **Selective reporting:** Occasional discrepancies were noted when studies described broader endpoints than those ultimately reported; however, the potential impact on overall conclusions was judged to be limited. |
| Certainty of evidence | 22 | Present assessments of certainty (or confidence) in the body of evidence for each outcome assessed. | **Certainty (Confidence) in the Body of Evidence**<br>• **EDC–Melatonin interactions on hormone receptor signaling:** Moderate confidence – multiple preclinical studies consistently show that melatonin can antagonize EDC-induced receptor dysregulation, with mechanistic plausibility via antioxidant and signaling pathway modulation.<br>• **EDC–Melatonin effects on metabolic endpoints (glucose, lipid, neuroendocrine markers):** Low–moderate confidence – fewer studies exist, but the observed protective effects of melatonin against EDC-induced metabolic disruption are directionally coherent.<br>• **EDC–Melatonin effects on reproductive outcomes:** Moderate confidence – melatonin attenuates EDC-associated disruptions in reproductive hormone activity and related endpoints across several animal models, though most outcomes are surrogate markers.<br>• **Clinical/translational outcomes in humans:** Very low confidence – direct human interventional or observational studies are lacking, representing a significant evidence gap. |
| **DISCUSSION** | | | |
| Discussion | 23a | Provide a general interpretation of the results in the context of other evidence. | **General Interpretation in the Context of Other Evidence**<br>Across the included studies, a consistent pattern emerges suggesting that melatonin can counteract or mitigate some of the endocrine-disrupting effects of chemicals such as bisphenol A, phthalates, and heavy metals. In animal and in vitro models, melatonin supplementation or signaling enhancement attenuated EDC-induced disruptions in hormone receptor activity (e.g., estrogen, androgen, and progesterone receptors), oxidative stress, and neuroendocrine endpoints. These findings align with broader evidence indicating melatonin's role as a pleiotropic regulator of circadian rhythms, antioxidant defense, and hormone homeostasis. Mechanistically, melatonin appears to modulate receptor-mediated pathways and downstream signaling nodes (e.g., AKT/FOXO1, MAPK) that EDCs typically dysregulate, while also reducing oxidative and inflammatory stress. |

| Section and Topic | Item # | Checklist item | Location where item is reported |
|---|---|---|---|
| | | | However, most evidence remains preclinical, and human data are sparse. Direct clinical studies evaluating melatonin's protective effects against EDC exposure on reproductive, metabolic, or neurobehavioral outcomes are limited. Moreover, outcome heterogeneity—ranging from receptor expression to systemic biomarkers—restricts direct comparison and translational interpretation. Nonetheless, the collective data support a model in which melatonin serves as an endogenous antagonist to EDC-induced endocrine disruption, providing a mechanistic basis for potential therapeutic or preventive applications. |
| | 23b | Discuss any limitations of the evidence included in the review. | **Limitations of the Evidence Base**<br>The current evidence is predominantly derived from animal and in vitro studies, often with small sample sizes and incomplete methodological reporting, including randomization, allocation concealment, and blinding, resulting in moderate risk of bias. Outcome measures were highly heterogeneous, encompassing hormonal assays, receptor expression, oxidative stress markers, behavioral endpoints, and systemic metabolic parameters, which limits cross-study comparability. Many studies relied on surrogate markers (e.g., melatonin receptor expression or EDC biomarker levels) rather than standardized physiological or clinical endpoints. Circadian timing, sex differences, and hormonal status were inconsistently accounted for, despite their known influence on melatonin signaling and EDC effects. Human data remain extremely limited, particularly interventional studies assessing melatonin's protective effects against EDC exposure on endocrine, metabolic, and neurobehavioral outcomes. These gaps constrain the direct translational relevance of current findings |
| | 23c | Discuss any limitations of the review processes used. | Limitations of the review processes<br>We did not register a protocol or apply GRADE, and no meta-analysis was feasible due to design/outcome heterogeneity; thus, effect sizes were not pooled, and publication bias statistics (e.g., funnel plots) were not applicable. Despite multi-database searches and reference chaining, residual reporting bias is possible (e.g., negative or null findings unpublished). Although dual-reviewer screening and extraction were used, unreported methodological details in primary studies constrained risk-of-bias judgments. Finally, the field's varied nomenclature/assays for orexin outcomes may have contributed to search sensitivity limits despite broad strategies. |
| | 23d | Discuss implications of the results for practice, policy, and future research. | **Implications for Practice, Policy, and Future Research**<br>**Practice/Clinical:** Current evidence supports the consideration of melatonin signaling when evaluating endocrine-disruptor–related physiological disturbances, including reproductive, metabolic, and neuroendocrine outcomes. Clinically, interventions targeting melatonin pathways |

| Section and Topic | Item # | Checklist item | Location where item is reported |
|---|---|---|---|
| | | | may have potential in mitigating EDC-induced disruptions in hormone-sensitive processes, though direct recommendations for patient care cannot yet be made due to limited human data.<br>**Policy/Guidelines:** Research policies and funding initiatives should prioritize studies assessing the protective role of melatonin against EDCs across multiple organ systems. Requirements for protocol preregistration, standardized outcome panels for melatonin and EDC biomarkers, and transparent reporting (including blinding and randomization) are critical to strengthen evidence quality and reproducibility.<br>**Future Research:**<br>• **Human interventional studies:** Conduct controlled trials or mechanistic studies evaluating melatonin supplementation or modulation in populations exposed to EDCs, measuring endocrine, metabolic, and neurophysiological endpoints.<br>• **Mechanistic mapping:** Investigate melatonin's antagonistic effects on EDC-mediated disruptions in hormone receptor signaling (e.g., ER, AR, PR) and downstream pathways across reproductive, metabolic, and neuroendocrine systems.<br>• **Standardization:** Develop harmonized assays for melatonin, EDC biomarkers, and relevant physiological endpoints; include timing (circadian) and sex-hormone status as control variables.<br>• **Translational bridges:** Evaluate whether melatonin-based interventions can mitigate EDC-induced dysregulation in human outcomes such as reproductive hormone balance, glucose metabolism, or neurobehavioral functions.<br>• **Regulatory focus:** Inform policies on safe EDC exposure levels, considering melatonin's potential protective role as a physiological countermeasure.<br>**Overall:** Evidence supports a modulatory role of melatonin against EDC-induced endocrine disruption, highlighting critical gaps in human translational studies and mechanistic understanding that should be prioritized in future research. |
| **OTHER INFORMATION** | | | |
| Registration and protocol | 24a | Provide registration information for the review, including the register name and registration number, or state that the review was not registered. | **Registration:** This review on melatonin as an antagonist of endocrine disruptors was not prospectively registered in a formal registry (e.g., PROSPERO) |
| | 24b | Indicate where the review protocol can be accessed, or | **Protocol Access:** No formal protocol was publicly archived prior to the initiation of this review on melatonin as an antagonist of endocrine disruptors. However, a detailed internal |

| Section and Topic | Item # | Checklist item | Location where item is reported |
|---|---|---|---|
| | | state that a protocol was not prepared. | methodological framework guiding the review process was developed and is available upon request from the corresponding author. |
| | 24c | Describe and explain any amendments to information provided at registration or in the protocol. | No deviations from the initial review objectives occurred. Minor refinements were made to the search strategy to include additional terminology related to endocrine-disrupting chemicals (EDCs), melatonin signaling pathways, and system-specific physiological endpoints. These refinements aimed to capture a broader range of studies on melatonin's antagonistic effects against EDCs across multiple organ systems. Importantly, these adjustments did not alter the original inclusion or exclusion criteria. |
| Support | 25 | Describe sources of financial or non-financial support for the review, and the role of the funders or sponsors in the review. | This review received no external funding. All research activities, including literature searches, study screening, data extraction, and synthesis, were conducted using institutional access and author resources. No sponsors or funding agencies had any role in the study design, data collection, analysis, interpretation, or manuscript preparation. |
| Competing interests | 26 | Declare any competing interests of review authors. | The authors declare no competing interests, financial or non-financial, related to this work. |
| Availability of data, code and other materials | 27 | Report which of the following are publicly available and where they can be found: template data collection forms; data extracted from included studies; data used for all analyses; analytic code; any other materials used in the review. | The following materials for this review on melatonin as an antagonist of endocrine disruptors **are** publicly available upon reasonable request to the corresponding author**:**<br>• Full-text **PDFs of included studies** (subject to copyright compliance).<br>• **SYRCLE's Risk of Bias** and **Cochrane RoB 2** assessments for each study.<br>• Completed **PRISMA 2020 checklist** for this review |

*From:* Page MJ, McKenzie JE, Bossuyt PM, Boutron I, Hoffmann TC, Mulrow CD, et al. The PRISMA 2020 statement: an updated guideline for reporting systematic reviews. BMJ 2021;372:n71. doi: 10.1136/bmj.n71. This work is licensed under CC BY 4.0. To view a copy of this license, visit https://creativecommons.org/licenses/by/4.0/
