# Supplementary material for: Melatonin as a protector against endocrine disruption across tissues: A systematic review
Source: Physiol Rep. 2026 Aug 10;14(15):e71049. doi: 10.14814/phy2.71049 (PMC13455548; doi:10.14814/phy2.71049)
Supplement: Supplementary file 2 [file PHY2-14-e71049-s002.pdf]

| Supplementary File S3: Risk of Bias Assessment report | | | | | | | | | | | |
|---|---|---|---|---|---|---|---|---|---|---|---|
| **No.** | **Study** | **Sequence Generation** | **Baseline Characteristics** | **Allocation Concealment** | **Random Housing** | **Blinding (Caregivers/ Researchers)** | **Blinding (Outcome Assessment)** | **Incomplete Outcome Data** | **Selective Outcome Reporting** | **Other Bias** | **Overall Risk** |
| 1 | Venditti et al., 2021a | Unclear | Low | Unclear | Low | Unclear | Unclear | Low | Low | Low | Some Concerns |
| 2 | Jiménez-Ortega et al., 2012 | Unclear | Low | Unclear | Low | Unclear | Unclear | Low | Low | Low | Some Concerns |
| 3 | Wang et al., 2024 | Unclear | Low | Unclear | Low | Unclear | Unclear | Low | Low | Low | Some Concerns |
| 4 | Kobroob et al., 2018 | Unclear | Low | Unclear | Low | Unclear | Unclear | Low | Low | Low | Some Concerns |
| 5 | Ebrahimi et al., 2021 | Unclear | Low | Unclear | Low | Unclear | Unclear | Low | Low | Low | Some Concerns |
| 6 | Bahrami et al., 2018 | Unclear | Low | Unclear | Low | Unclear | Unclear | Low | Low | Low | Some Concerns |
| 7 | Venditti et al., 2021b | Unclear | Low | Unclear | Low | Unclear | Unclear | Low | Low | Low | Some Concerns |
| 8 | T.-N. Yang et al., 2024 | Unclear | Low | Unclear | Low | Unclear | Unclear | Low | Low | Low | Some Concerns |
| 9 | Gładysz et al., 2023 | Unclear | Low | Unclear | Low | Unclear | Unclear | Low | Low | Low | Some Concerns |

| | | | | | | | | | | | |
|---|---|---|---|---|---|---|---|---|---|---|---|
| 10 | Liu et al., 2019 | Unclear | Low | Unclear | Low | Unclear | Unclear | Low | Low | Low | Some Concerns |
| 11 | Poliandri et al., 2006 | Unclear | Low | Unclear | Low | Unclear | Unclear | Low | Low | Low | Some Concerns |
| 12 | Xia et al., 2024 | Unclear | Low | Unclear | Low | Unclear | Unclear | Low | Low | Low | Some Concerns |
| 13 | Chouchene et al., 2025 | Unclear | Low | Unclear | Low | Unclear | Unclear | Low | Low | Low | Some Concerns |
| 14 | Flora et al., 2004 | Unclear | Low | Unclear | Low | Unclear | Unclear | Low | Low | Low | Some Concerns |
| 15 | Ishtiaq et al., 2021 | Unclear | Low | Unclear | Low | Unclear | Unclear | Low | Low | Low | Some Concerns |
| 16 | Tabassum et al., 2017 | Unclear | Low | Unclear | Low | Unclear | Unclear | Low | Low | Low | Some Concerns |
| 17 | Xu et al., 2021 | Unclear | Low | Unclear | Low | Unclear | Unclear | Low | Low | Low | Some Concerns |
| 18 | S. Yang et al., 2024 | Unclear | Low | Unclear | Low | Unclear | Unclear | Low | Low | Low | Some Concerns |
| 19 | Anjum et al., 2011 | Unclear | Low | Unclear | Low | Unclear | Unclear | Low | Low | Low | Some Concerns |
| 20 | Wu et al., 2013 | Unclear | Low | Unclear | Low | Unclear | Unclear | Low | Low | Low | Some Concerns |
| 21 | Olukole et al., 2018 | Unclear | Low | Unclear | Low | Unclear | Unclear | Low | Low | Low | Some Concerns |
| 22 | Abdullah and | Unclear | Low | Unclear | Low | Unclear | Unclear | Low | Low | Low | Some Concerns |

| | | | | | | | | | | | |
|---|---|---|---|---|---|---|---|---|---|---|---|
| | Rashid, 2020 | | | | | | | | | | |
| 23 | Lamtai et al., 2021 | Unclear | Low | Unclear | Low | Unclear | Unclear | Low | Low | Low | Some Concerns |
| 24 | Kumar et al., 2021 | Unclear | Low | Unclear | Low | Unclear | Unclear | Low | Low | Low | Some Concerns |
| 25 | Liu et al., 2015 | Unclear | Low | Unclear | Low | Unclear | Unclear | Low | Low | Low | Some Concerns |
| 26 | El-Missiry et al., 2014 | Unclear | Low | Unclear | Low | Unclear | Unclear | Low | Low | Low | Some Concerns |
| 27 | Shi et al., 2025 | Unclear | Low | Unclear | Low | Unclear | Unclear | Low | Low | Low | Some Concerns |
| 28 | Olukole et al., 2019 | Unclear | Low | Unclear | Low | Unclear | Unclear | Low | Low | Low | Some Concerns |
| 29 | Farag et al., 2024 | Unclear | Low | Unclear | Low | Unclear | Unclear | Low | Low | Low | Some Concerns |
| 30 | Zhao et al., 2023 | Unclear | Low | Unclear | Low | Unclear | Unclear | Low | Low | Low | Some Concerns |
| 31 | Zhang et al., 2019 | Unclear | Low | Unclear | Low | Unclear | Unclear | Low | Low | Low | Some Concerns |
| 32 | Gül et al., 2018 | Unclear | Low | Unclear | Low | Unclear | Unclear | Low | Low | Low | Some Concerns |
| 33 | Şensoy, 2024 | Unclear | Low | Unclear | Low | Unclear | Unclear | Low | Low | Low | Some Concerns |
| 34 | Guohua et al., 2021 | Unclear | Low | Unclear | Low | Unclear | Unclear | Low | Low | Low | Some Concerns |
| 35 | Zhang et al., 2017 | Unclear | Low | Unclear | Low | Unclear | Unclear | Low | Low | Low | Some Concerns |

| | | | | | | | | | | | |
|---|---|---|---|---|---|---|---|---|---|---|---|
| 36 | Zhang et al., 2018 | Unclear | Low | Unclear | Low | Unclear | Unclear | Low | Low | Low | Some Concerns |
| 37 | Lin et al., 2025 | Unclear | Low | Unclear | Low | Unclear | Unclear | Low | Low | Low | Some Concerns |
| 38 | Omeiza et al., 2021 | Unclear | Low | Unclear | Low | Unclear | Unclear | Low | Low | Low | Some Concerns |
| 39 | Turker Yavuzaslan et al., 2024 | Unclear | Low | Unclear | Low | Unclear | Unclear | Low | Low | Low | Some Concerns |
| 40 | Wahby et al., 2017 | Unclear | Low | Unclear | Low | Unclear | Unclear | Low | Low | Low | Some Concerns |
| 41 | Lamtai et al., 2020 | Unclear | Low | Unclear | Low | Unclear | Unclear | Low | Low | Low | Some Concerns |
| 42 | Yao et al., 2023 | Unclear | Low | Unclear | Low | Unclear | Unclear | Low | Low | Low | Some Concerns |
| 43 | Sousa et al., 1997 | Unclear | Low | Unclear | Low | Unclear | Unclear | Low | Low | Low | Some Concerns |
| 44 | Yang et al., 2021 | Unclear | Low | Unclear | Low | Unclear | Unclear | Low | Low | Low | Some Concerns |
| 45 | Sarihan et al., 2015 | Unclear | Low | Unclear | Low | Unclear | Unclear | Low | Low | Low | Some Concerns |
| 46 | Rao and Chhunchha, 2010 | Unclear | Low | Unclear | Low | Unclear | Unclear | Low | Low | Low | Some Concerns |
| 47 | Karaoz et al., 2002 | Unclear | Low | Unclear | Low | Unclear | Unclear | Low | Low | Low | Some Concerns |
| 48 | Kadir et al., 2024 | Unclear | Low | Unclear | Low | Unclear | Unclear | Low | Low | Low | Some Concerns |
| 49 | Heena Tabassum | Unclear | Low | Unclear | Low | Unclear | Unclear | Low | Low | Low | Some Concerns |

| | | | | | | | | | | | |
|---|---|---|---|---|---|---|---|---|---|---|---|
| | et al., 2017 | | | | | | | | | | |
| 50 | Sun et al., 2022 | Unclear | Low | Unclear | Low | Unclear | Unclear | Low | Low | Low | Some Concerns |
| 51 | Bali et al., 2016 | Unclear | Low | Unclear | Low | Unclear | Unclear | Low | Low | Low | Some Concerns |
| 52 | Akçay et al., 2020 | Unclear | Low | Unclear | Low | Unclear | Unclear | Low | Low | Low | Some Concerns |
| 53 | Goktepe et al., 2023 | Unclear | Low | Unclear | Low | Unclear | Unclear | Low | Low | Low | Some Concerns |
| 54 | Shaker and Al-Wasiti, 2023 | Unclear | Low | Unclear | Low | Unclear | Unclear | Low | Low | Low | Some Concerns |
| 55 | Stacchiotti et al., 2006 | Unclear | Low | Unclear | Low | Unclear | Unclear | Low | Low | Low | Some Concerns |
| 56 | Anjuma et al.,2011 | Unclear | Low | Unclear | Unclear | Unclear | Unclear | Low | Low | Low | Some Concerns |
| 57 | Oluwasola et al., 2026 | Unclear | Low | Unclear | Unclear | Unclear | Unclear | Low | Low | Low | Some Concerns |
| 58 | Alagbonsi & Olayaki, 2018 | Unclear | Low | Unclear | Unclear | Unclear | Unclear | Low | Low | Low | Some Concerns |
| 59 | Alagbonsi et al., 2016 | Unclear | Low | Unclear | Unclear | Unclear | Unclear | Low | Low | Low | Some Concerns |

| | | | | | | | | | | | |
|---|---|---|---|---|---|---|---|---|---|---|---|
| 60 | Alagbonsi et al., 2018 | Unclear | Low | Unclear | Unclear | Unclear | Unclear | Low | Low | Low | Some Concerns |
| 61 | Alagbonsi et al., 2015 | Unclear | Low | Unclear | Unclear | Unclear | Unclear | Low | Low | Low | Some Concerns |
