# Supplementary material for: Melatonin as a protector against endocrine disruption across tissues: A systematic review
Source: Physiol Rep. 2026 Aug 10;14(15):e71049. doi: 10.14814/phy2.71049 (PMC13455548; doi:10.14814/phy2.71049)
Supplement: Supplementary file 3 [file PHY2-14-e71049-s003.pdf]

Supplementary File S2: Search Strings used for each database

Database	Search String (Executed Exactly As Shown)	Date Run
**PubMed / MEDLINE**	("Melatonin"[Mesh] OR melatonin[tiab] OR "N-acetyl-5-methoxytryptamine"[tiab] OR pineal[tiab] OR "MT1 receptor"[tiab] OR "MT2 receptor"[tiab]) AND ("Endocrine Disruptors"[Mesh] OR "Environmental Pollutants"[Mesh] OR endocrine disrupt*[tiab] OR EDC[tiab] OR xenoestrogen*[tiab] OR bisphenol*[tiab] OR BPA[tiab] OR phthalate*[tiab] OR "polychlorinated biphenyl*"[tiab] OR PCB[tiab] OR "polybrominated diphenyl ether*"[tiab] OR PBDE[tiab] OR perfluor*[tiab] OR PFAS[tiab] OR pesticide*[tiab] OR "heavy metal*"[tiab] OR organochlorine[tiab] OR "flame retardant*"[tiab] OR plasticizer*[tiab]) AND (thyroid*[tiab] OR reproductive[tiab] OR ovarian[tiab] OR testicular[tiab] OR adrenal[tiab] OR pancreatic[tiab] OR hepatic[tiab] OR neuroendocrine[tiab] OR placenta*[tiab]) AND ("Animals"[Mesh] OR "Models, Animal"[Mesh] OR "Cells"[Mesh] OR "Humans"[Mesh] OR rat[tiab] OR rats[tiab] OR mouse[tiab] OR mice[tiab] OR rodent*[tiab] OR "in vitro"[tiab] OR "in vivo"[tiab] OR experimental[tiab] OR "cell culture"[tiab]) AND ("2000/01/01"[Date - Publication] : "2025/10/31"[Date - Publication]) AND (english[Language])	01/02/2026
**Wiley Online Library**	All Fields: (melatonin OR "N-acetyl-5-methoxytryptamine" OR pineal OR "MT1 receptor" OR "MT2 receptor") AND All Fields: ("endocrine disrupt*" OR EDC OR bisphenol* OR BPA OR phthalate* OR "polychlorinated biphenyl*" OR PCB OR "polybrominated diphenyl ether*" OR PBDE OR perfluor* OR PFAS OR pesticide* OR "heavy metal*" OR organochlorine OR "flame retardant*" OR plasticizer*) AND All Fields: (thyroid* OR reproductive OR ovarian OR testicular OR adrenal OR pancreatic OR hepatic OR neuroendocrine OR placenta*) AND All Fields: (rat OR mouse OR rodent* OR "in vitro" OR "in vivo" OR experimental OR "cell culture") Filters: Published between Jan 2000 and Oct 2025; English	01/02/2026
**Web of Science**	TITLE-ABS-KEY (melatonin OR "N-acetyl-5-methoxytryptamine" OR pineal OR "MT1 receptor" OR "MT2 receptor") AND TITLE-ABS-KEY ("endocrine disrupt*" OR edc OR xenoestrogen* OR bisphenol* OR bpa OR phthalate* OR "polychlorinated biphenyl*" OR pcb OR "polybrominated diphenyl ether*" OR pbde OR perfluor* OR pfas OR pesticide* OR "heavy metal*" OR organochlorine OR "flame retardant*" OR plasticizer*) AND TITLE-ABS-KEY (thyroid* OR reproductive OR ovarian OR testicular OR adrenal OR pancreatic OR hepatic OR neuroendocrine OR placenta*) AND TITLE-ABS-KEY (rat OR rats OR mouse OR mice OR rodent* OR "in vitro" OR "in vivo" OR experimental OR "cell culture") AND PUBYEAR > 1999 AND PUBYEAR < 2026 AND (LIMIT-TO (LANGUAGE , "English"))	01/02/2026
